# Novel interactions between the C5‐C5aR1 Axis and IF1: Implications for kidney mitochondrial physiology and ischemia–reperfusion injury

**DOI:** 10.14814/phy2.70942

**Published:** 2026-05-29

**Authors:** Madison McGraw, Amod Sharma, Dinesh Bhattarai, Neriman Gokden, LeeAnn Macmillan‐Crow, Nirmala Parajuli

**Affiliations:** ^1^ Department of Pharmacology & Toxicology University of Arkansas for Medical Sciences Little Rock Arkansas USA; ^2^ Department of Pathology University of Arkansas for Medical Sciences Little Rock Arkansas USA

**Keywords:** ATPase inhibitory factor 1, Avacopan, complement 5, kidney injury, mitochondria

## Abstract

Ischemia‐reperfusion injury (IRI) is a prevalent condition that predominantly afflicts hospitalized patients, inducing acute kidney injury (AKI). In recent years, complement 5 (C5) and its anaphylatoxin receptor C5aR1 have been implicated in driving kidney IRI and loss of function. Beyond this, prior studies suggest C5‐C5aR1 mediates mitochondrial ROS production, although its role in the mitochondria has never been fully characterized. Here, we leverage a previously generated model of C5 gene deletion (male C5^−/−^ rats) and the clinically relevant C5aR1 inhibitor Avacopan (AV) to investigate C5‐C5aR1 signaling in renal mitochondrial physiology and pathophysiology. For the first time, we report that C5‐C5aR1 axis inhibition modifies physiological mitochondrial protein levels, respiratory activity, and complexes/supercomplexes. We identified a novel relationship between the C5‐C5aR1 axis and ATPase Inhibitory Factor 1 (IF1), a potent regulator of the ATP synthase, using in vivo and in vitro approaches. Post‐IRI, C5‐C5aR1 axis inhibition improved kidney function/morphology and preserved ATP levels, despite IRI‐mediated disintegration of mitochondrial complexes and supercomplexes. We show in vitro that C5‐C5aR1 axis inhibition facilitated IF1‐dependent ATP recovery via the glycolysis pathway. Collectively, our results demonstrate a complex interplay between C5‐C5aR1 and IF1 in renal mitochondria, which contributes to mitochondrial pathophysiology during IRI.

## 1 INTRODUCTION

End‐stage kidney disease (ESKD) currently affects over 800,000 patients in the United States and accounts for 7%–8% of total Medicare spending despite comprising only 1%–2% of all Medicare beneficiaries (*Annual Data Report*, 2026). This is largely attributable to the lack of treatment options available to ESKD patients, who rely on cost‐intensive dialysis therapies until kidney replacement therapy can be initiated (CDC, 2025; Kidney Disease Statistics for the United States—NIDDK, 2026). As ESKD is an advanced stage of disease, one of the predominant predictive factors for the development of chronic kidney disease (CKD) progressing to ESKD is a prior acute kidney injury, or AKI. AKI is common, affecting over 13 million people and resulting in 1.7 million deaths/year worldwide (Abebe et al., 2021; Institute for Healthcare Policy and Innovation, 2026). Furthermore, the severity of AKI is explicitly linked to the risk of CKD progression (Chawla et al., 2011). Although the causes of AKI are multifactorial, it most commonly occurs in hospitalized patients and therefore may be preventable or even reversible with the correct management (Kate et al., 2020). Kidney replacement therapy, for example, carries a risk of inducing a subtype of AKI termed ischemia–reperfusion injury (IRI) (Debout et al., 2015; Nieuwenhuijs‐Moeke et al., 2020). Renal IRI occurs when areas of the kidney tissue receive inadequate blood flow, resulting in a dearth of oxygen and nutrients that leads to cell death. If the blood supply is later restored, the resulting reperfusion of the affected tissue paradoxically causes further damage and drives pro‐inflammatory pathologies (Guerrero‐Mauvecin et al., 2023; Li et al., 2024; Linkermann et al., 2013). Other clinical scenarios at risk for IRI include significant hypotension, myocardial infarction, embolism, stroke, or planned surgical procedures resulting in blood loss (Hoste et al., 2006; Malek & Nematbakhsh, 2015). In the kidney, IRI is particularly dangerous due to the high risk of developing chronic kidney disease (CKD), a long‐term condition that may progress to ESKD (Dong et al., 2019; Yang et al., 2024). Significantly, no clear druggable targets have been established for the treatment of renal IRI despite its prevalence. However, because renal IRI is driven by a pro‐inflammatory pathology (Guerrero‐Mauvecin et al., 2023; Nieuwenhuijs‐Moeke et al., 2020; Peng et al., 2019), various factors including complement proteins have been identified that may hold therapeutic promise.

The complement system is a network of 50+ proteins that circulate in the serum as a component of innate immunity. Over the years, this system has been associated with diverse functions and pathologies, from specific complement‐mediated diseases (Smith et al., 2019; Stern & Connell, 2019) to autoimmune disorders (Chighizola et al., 2020; Sadik et al., 2018) and neurodegenerative disease (Cole et al., 2006; Dalakas et al., 2020; Krance et al., 2021). In the kidney, excessive complement activation contributes to IRI severity (Bartoszek et al., 2019; Hu et al., 2018; Pratt et al., 2003; Thurman et al., 2006). While complement can be activated through three distinct pathways, prior studies implicate alternative pathway activation as one of the main drivers of renal IRI (Bartoszek et al., 2019; Casiraghi et al., 2017; Thurman et al., 2003; Zheng et al., 2006). The alternative pathway can be triggered by the autohydrolysis of complement 3 (C3) into C3a and C3b, which leads to the formation of a key C5 convertase enzyme. This enzyme cleaves complement 5 (C5) into subcomponents C5a and C5b. While C5b is critical to forming the membrane attack complex (MAC), the endpoint of the complement cascade responsible for lysing cells, the C5a product carries out a variety of functions via receptor interactions (Zhou et al., 2000). Most notably, C5a binds to C5aR1 on the surface of immune cells to stimulate chemotaxis (Cravedi et al., 2013; Kovtun et al., 2017; Sadik et al., 2018). However, emerging studies examining the C5a‐C5aR1 axis support an intracellular role that has yet to be fully explored (Bröker et al., 2018; Ding et al., 2022; Pandey et al., 2020).

Our group recently demonstrated, in a rodent model of renal IRI, that C5 acts as a critical mediator of renal tissue damage and function loss (McGraw et al., 2025). These results align with previous studies highlighting the therapeutic potential of targeting C5 and C5aR1 during renal IRI (Adams et al., 2021; Arumugam et al., 2003; Ishigooka et al., 2022; Peng et al., 2012), further emphasizing the need to elucidate C5's role in the kidney. Although the liver remains the primary site of complement synthesis, various extrahepatic tissues, including the kidney, produce complement proteins intracellularly (Molinari et al., 2025; Timmerman et al., 1996; Zhou et al., 2001). Under cellular stress, intracellular complement acts as a critical, noncanonical mediator of metabolic adaptation and survival (Lin et al., 2024; Ratajczak et al., 2025). Local synthesis of complement occurs predominantly within immune cells (Laufer et al., 2001) and epithelial/endothelial cell types (Arbore et al., 2017; Khanal, 2022), including renal proximal tubular cells (Brooimans et al., 1991; Lo et al., 2021; Seelen et al., 1993; Timmerman et al., 1996; Welch et al., 1993) which are susceptible to IRI. Despite the prevalence of C5 in the kidney (both circulating and locally synthesized) the C5 protein's contribution to renal tissue damage and loss of function during IRI is not completely understood.

Beyond C5's known role as a mediator of local inflammatory injury (Farrar et al., 2006; Pratt et al., 2002; Tang et al., 1999), recent studies suggest that the intracellular complement proteins are key regulators of cellular metabolism (Hess & Kemper, 2016; Ishii et al., 2021; Thapa et al., 2023). The expression of complement anaphylatoxin receptor C5aR1 has been identified on the outer membrane of the major organelle associated with metabolism, the mitochondria (Ishii et al., 2021; Niyonzima et al., 2021; Rahman et al., 2021). Stimulation of C5aR1 is documented to stimulate respiratory burst and reactive oxygen species (ROS) production in several immune cell subtypes (Silva et al., 2023; Arbore et al., 2016; Bröker et al., 2018; Niyonzima et al., 2021), but its role in kidney mitochondria is yet unexplored. Loss of mitochondrial function is a well‐documented consequence of renal IRI (Huang et al., 2024; Loor et al., 2011; Pabla & Bajwa, 2022) that undercuts tissue repair mechanisms (Kazeminia & Eirin, 2024) and contributes to tissue injury through the formation of mitochondria‐derived ROS (Abebe et al., 2021; Nieuwenhuijs‐Moeke et al., 2020). Thus, there is a critical need to identify factors that sensitize renal mitochondria to damage and dysfunction following IRI.

Disruption of the electron transport chain (ETC) is a clear indicator for mitochondrial dysfunction. Not only does the loss of ETC function often result in deficient ATP, but the ROS produced as a normal part of aerobic respiration also increase. Prior studies have shown that mitochondria‐targeted treatments, such as antioxidants, can suppress complement proteins and mitigate ischemic injury (Yang et al., 2013). Conversely, Torp. et al. recently reported that complement C3 deficiency resulted in reduced mitochondrial respiration in mice (Torp et al., 2022). Similarly, administration of the C5 cleaved anaphylatoxin C5a activated C5aR1 and promoted ROS production as well as mitochondria‐dependent apoptosis in kidney endothelial cells (Tsai et al., 2019). These previous studies suggest that C5–C5aR1 may have some impact on ETC function, although the specifics of such an interaction are unknown.

One well‐established factor that regulates ETC function is the organization of the mitochondrial complexes, which may form supramolecular assemblies with each other to enhance ETC efficiency (Guan et al., 2022; Javadov et al., 2021). The formation of these so‐called “supercomplexes” maximizes ATP production while minimizing ROS (Lapuente‐Brun et al., 2025). Disintegration of supercomplexes, a documented phenomenon during IRI, contributes to pathogenesis by handicapping ATP synthesis and mediating increased ROS (Jang et al., 2017). However, the organization of ETC complexes into supercomplexes is far from the only parameter governing ETC function and ATP. For example, IF1 (ATPase Inhibitory Factor 1) is a nuclear‐encoded protein that was initially discovered as an inhibitor of ATP hydrolysis (Acin‐Perez et al., 2023). In acidic conditions, IF1 proteins form active dimers that bind to the catalytic component of the ATP synthase (Boreikaite et al., 2019; Gu et al., 2019). Once bound, the IF1 dimer is a regulator of ATP synthesis and hydrolysis which responds to the proton gradient (Sgarbi et al., 2018). Tangentially, several prior studies have indicated that IF1 is a potent driver of aerobic glycolysis (Formentini et al., 2012; Sánchez‐Cenizo et al., 2010; Zhou et al., 2022) as a means of ATP production, which supports cellular survival in the event of a lack of oxygen. As emerging insights into C5‐C5aR1 in the mitochondria increasingly implicate a role in ETC function, a thorough investigation into an effect on key regulatory factors is warranted.

In this study, we sought to investigate the role of the C5‐C5aR1 axis in renal and tubular cell mitochondrial function at baseline. Subsequently, we aimed to clarify C5‐C5aR1's contribution to mitochondrial dysfunction and renal injury following IRI. We hypothesized that targeting this axis would stabilize ETC complexes, promote supercomplex assembly, and enhance ETC efficiency, thereby mitigating mitochondrial dysfunction after renal IRI. To test this hypothesis, we utilized a newly generated C5^−/−^ rat model validated in our recent study (McGraw et al., 2025) to mimic C5 deficiency, along with the clinically relevant C5aR1 inhibitor Avacopan (AV) to isolate C5aR1‐specific effects (Garg & Frishman, 2023). Additionally, we employed an in vitro model of normal rat kidney proximal tubular epithelial (NRK) cells treated with C5 siRNA or Avacopan to examine the role of the intracellular C5‐C5aR1 axis. By inhibiting C5‐C5aR1 in vitro, the role of the intracellular C5‐C5aR1 axis could be delineated from the contributions of extracellular C5 (i.e. circulating C5 synthesized by the liver). Through these experiments, we uncovered a previously unrecognized relationship between the C5‐C5aR1 axis and IF1, the master regulator of ATP synthase catalytic activity (Arbore et al., 2016) and a known mediator of aerobic glycolysis (Sgarbi et al., 2018; Zhou et al., 2022). Together these findings identify a previously unrecognized role for the C5‐C5aR1 axis, acting in concert with IF1, as a metabolic regulatory pathway that stabilizes mitochondrial function, supports ATP restoration, and confers protection against renal IRI.

## 2 RESULTS

### 2.1 Avacopan improves kidney function and ameliorates tissue injury post‐IRI

C5 activation is a hallmark of IRI and produces two cleaved fragments, C5a and C5b. While the C5a fragment binds to the C5aR1 receptor to drive effector functions, C5b contributes to MAC assembly and cell lysis. To isolate the impact of C5a‐C5aR1 on kidney function and tissue morphology post‐IRI, we pretreated rats with Avacopan (AV; 30 mg/kg, 1 h prior to ischemia). AV is a small molecule, reversible C5aR1 antagonist currently FDA‐approved for severe ANCA‐associated vasculitis (Tavneospro, 2023).

**FIGURE 1:**
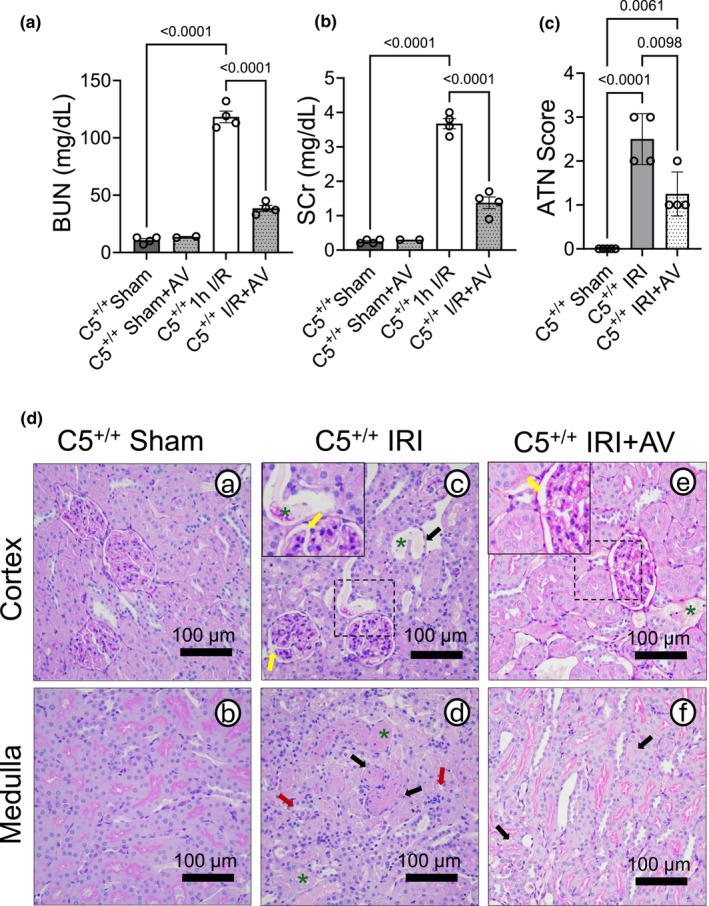
Avacopan (AV) improves kidney function and mitigates tubular necrosis after ischemia and reperfusion (IRI). Wild type (C5^+/+^) rats (male) were treated with 30 mg/kg AV i.p. 1 h prior to surgery. Animals were exposed to bilateral renal ischemia (1 h) plus reperfusion (24 h) to induce IRI. Sham surgery (right nephrectomy) served as a control. Whole blood and kidneys were removed post‐IRI and used for downstream analysis. (a, b) Bar graphs showing blood kidney function measurements. (a) Blood urea nitrogen (BUN) and (b) serum creatinine (SCr) were quantified using the VetScan i‐STAT system. Data are shown as the mean +/− SD, *n* = 2*‐4. *2 representative C5^+/+^+AV Sham controls are included for comparison against C5^+/+^ Shams. (c) Bar graph showing the acute tubular necrosis (ATN) score, quantified by a licensed pathologist. Data are shown as the mean +/− SD, *n* = 4. (d) Representative micrographs (*n* = 4 per group) from the kidney cortex and medulla stained with PAS.

As expected due to the results of our prior C5 knockout (C5^−/−^) study (McGraw et al., 2025), the blood urea nitrogen (BUN) and serum creatinine (SCr) were significantly elevated in vehicle‐treated rats after IRI (C5^+/+^ 1 h I/R), which was blunted by AV‐treatment (C5^+/+^ I/R + AV) (Figure 1a,b). Given the improved kidney function by AV post IRI, we next assessed kidney histopathology. Acute tubular necrosis (ATN) was scored by a blinded pathologist. As expected, the C5^+/+^ sham group displayed minimal tissue injury (all scored 0), whereas the untreated IRI group developed prominent necrotic lesions (Figure 1c). As expected, AV treatment attenuated necrosis (Figure 1c), consistent with our prior findings in C5^−/−^ rats post‐IRI (McGraw et al., 2025). Representative PAS images from C5^+/+^ sham rats show normal kidney morphology (Figure 1d). Post‐IRI, the hallmark features of IRI were evident—including tubular casts (green asterisks), brush border loss (black arrows), cell swelling/blebbing (red arrows), and mesangial widening (yellow arrows) (Figure 1c). Notably, AV‐treated rats displayed fewer morphological disruptions than vehicle‐treated rats post‐IRI (Figure 1c). Together these findings indicate that the C5a‐C5aR1 axis significantly contributes to the decline of kidney function postinjury, independent of C5b/MAC.

### 2.2 Avacopan attenuates kidney injury biomarker deposition

**FIGURE 2:**
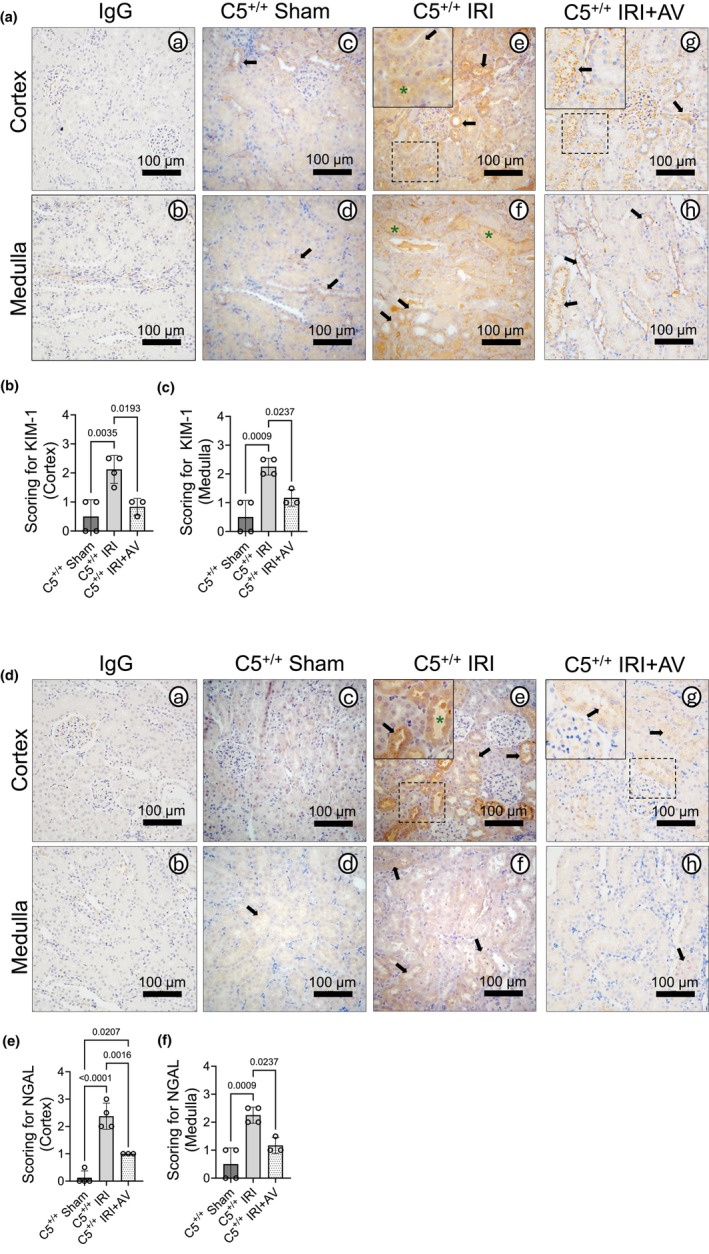
Avacopan attenuates kidney injury biomarker deposition post‐IRI. Wild‐type (C5^+/+^) rats (male) were dosed with 30 mg/kg AV i.p. 1 h prior to IRI surgery. Animals underwent bilateral renal ischemia for 1 h plus 24 h of reperfusion. Animals undergoing sham procedures (right nephrectomy) served as controls. Kidneys were isolated post‐IRI and processed for immunohistochemical analysis. (a) Representative micrographs (*n* = 4 per group) of formalin‐fixed kidney sections stained for KIM‐1. Black arrows indicate KIM‐1 deposition in tubular/peritubular regions and green asterisks indicate intratubular deposition. (b, c) Graphs showing semi‐quantitative analysis of KIM‐1 deposition in the (b) kidney cortex and (c) medulla across experimental groups. Data are expressed as the mean +/− SD, *n* = 4. (d) Representative micrographs (*n* = 4 per group) of formalin‐fixed kidney sections immunohistochemically stained for NGAL. Black arrows indicate tubular/peritubular deposition of NGAL, and green asterisks indicate intratubular deposition. (e, f) Graphs showing the semi‐quantitative analysis of NGAL staining in the (e) cortex and (f) medulla regions across experimental groups. Data are shown as the mean +/− SD, *n* = 4.

Kidney injury biomarkers such as Kidney Injury Molecule‐1 (KIM‐1) (Bonventre, 2014) and Neutrophil Gelatinase‐Associated Lipocalin (NGAL) (Romejko et al., 2023) are early clinical biomarkers of kidney injury. Compared to isotype‐matched controls (Figure 2a), C5^+/+^ sham rats exhibited a low level of KIM‐1 in renal tubules (Figure 2a; black arrows). As expected, IRI increased tubular KIM‐1 (Figure 2a; black arrows) and induced luminal sloughing (green asterisks). Compared to the untreated group, AV‐treated rats displayed localized KIM‐1 primarily on intralumenal surfaces (Figure 2a; black arrows) and decreased sloughing. Semi‐quantitative scoring confirmed increased cortical (Figure 2b) and medullary (Figure 2c) KIM‐1 in C5^+/+^ rats after IRI that was attenuated by AV treatment.

Similarly, NGAL showed a low‐level tubular signal in C5^+/+^ shams compared to isotype‐matched controls (Figure 2d; black arrows). After IRI, NGAL was increased in renal tubular cells (Figure 2d; black arrows) with luminal deposition in C5^+/+^ kidneys (green asterisks), which was reduced by AV treatment (Figure 2d; black arrows). Semi‐quantitative scoring mirrored KIM‐1 patterns (Figure 2e,f). Together, these data recapitulate the protective effect of C5aR1 inhibition, which attenuates renal tissue injury due to IRI.

### 2.3 Effect of C5‐C5aR1 on overall necrosis, apoptosis, and Nitrotyrosine in the kidney post‐IRI

**FIGURE 3:**
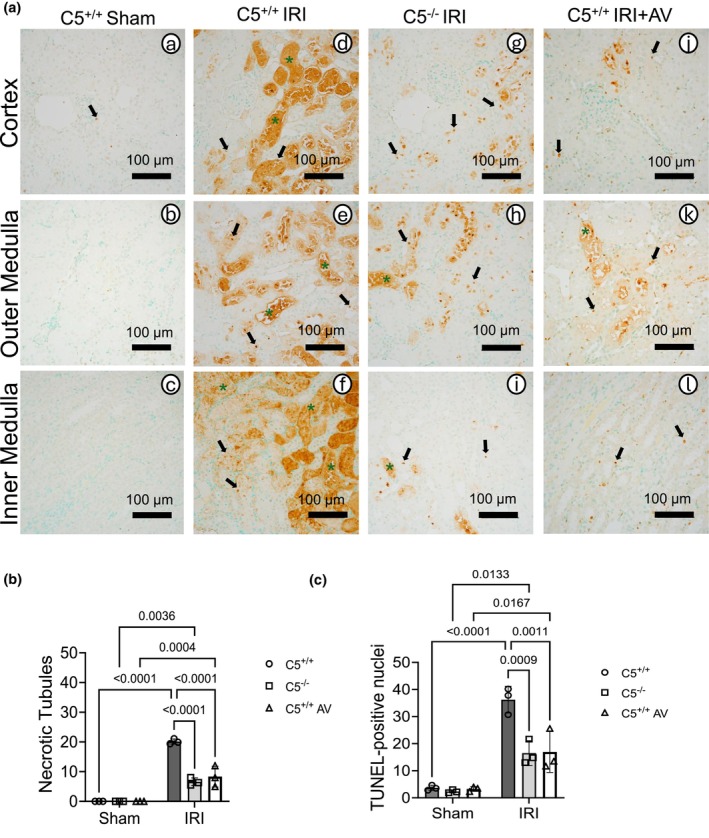
C5^−/−^ and Avacopan treatment alleviate IRI‐induced apoptosis and necrosis in kidneys. Male wild‐type (C5^+/+^) and homozygous C5 knockout (C5^−/−^) rats were exposed to a surgical model of IRI consisting of bilateral renal ischemia (1 h) and 24 h reperfusion. A group of C5^+/+^ rats were dosed with 30 mg/kg AV i.p. for C5aR1 inhibition 1 h prior to the surgery. Sham surgeries (right nephrectomy) served as controls for all experimental groups. Kidneys were isolated after IRI and processed for analysis of apoptosis and necrosis using the TUNEL method. (a) Representative micrographs (*n* = 3–4 per group) from formalin‐fixed and paraffin‐embedded kidney sections are shown. Black arrows indicate cells with TUNEL‐positive nuclei and green asterisks indicate TUNEL‐positive necrotic tubules. (b, c) Graphs showing semi‐quantitative counts of (b) necrotic tubules and (c) TUNEL‐positive nuclei across all experimental groups. Data are expressed as the mean +/− SD, *n* = 3–4.

During IRI, tubular cell death occurs predominantly via two major mechanisms—apoptosis and necrosis. Whereas apoptosis constitutes a controlled process that minimizes the release of cellular contents into the extracellular space, necrosis involves the acute release of intracellular contents and provokes inflammation (Bertheloot et al., 2021). We investigated the impact of two interventions, namely C5^−/−^ and AV treatment, on apoptosis and necrosis post‐IRI using the in‐situ terminal transferase‐mediated dUTP nick‐end labeling (TUNEL) method (Figure 3). C5^+/+^ sham kidneys showed few apoptotic cells (Figure 3a; black arrows) and no necrotic cells, reflecting a physiologically healthy turnover of tubular cells. After IRI, C5^+/+^ rats displayed increased apoptotic cells (Figure 3a; black arrows) and necrotic tubules (green asterisks). Both C5^−/−^ (Figure 3a) and AV‐treated rats (Figure 3a j‐l) exhibited significantly fewer apoptotic cells (black arrows) and necrotic tubules (green asterisks) post‐IRI, as quantified in Figure 3b,c. This indicates that the C5‐C5aR1 axis contributes to both necrosis and apoptosis following IRI.

**FIGURE 4:**
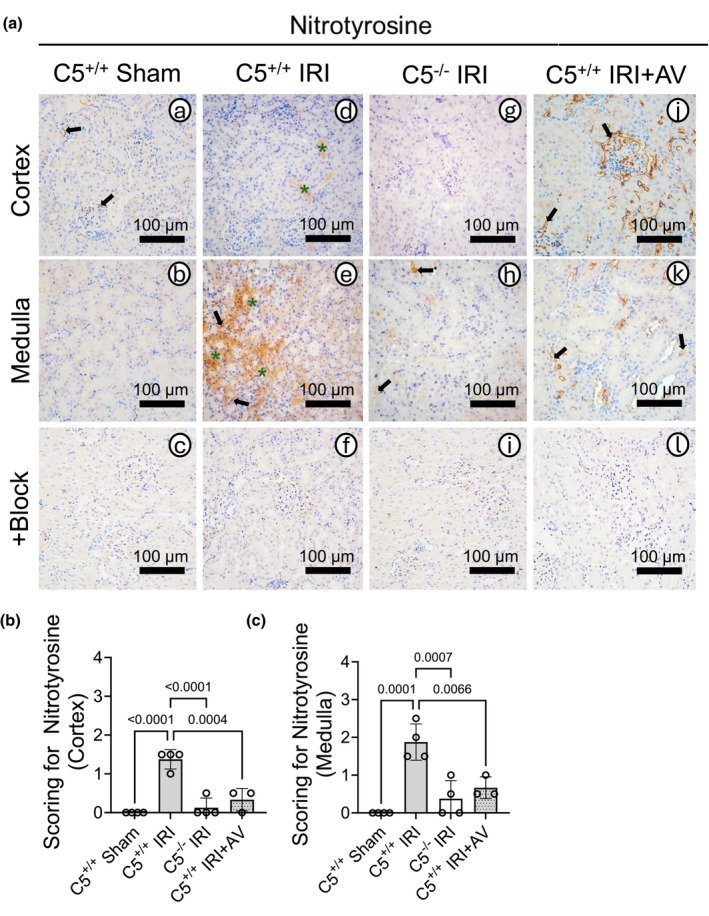
C5‐C5aR1 exacerbates nitrotyrosine residue formation after renal IRI. Male wild‐type (C5^+/+^) and homozygous (C5^−/−^) rats were exposed to a surgical IRI model of 1 h bilateral renal ischemia and 24 h reperfusion. To isolate C5aR1‐specific effects, a group of C5^+/+^ rats were dosed with 30 mg/kg AV i.p. 1 h prior to surgery. Sham surgery (right nephrectomy) was performed to serve as a control. Kidneys were isolated after IRI and processed for immunohistochemical analysis. (a) Representative micrographs (*n* = 3–4 per group) of formalin‐fixed kidney sections stained for nitrotyrosine or nitrotyrosine plus block (Figure 4a c,f,i,l). Black arrows indicate tubular/peritubular nitrotyrosine staining, and green asterisks denote intratubular staining. (b, c) Graphs showing the semi‐quantitative analysis of nitrotyrosine staining in the (b) kidney cortex and (c) medulla regions across experimental groups. Data are shown as the mean +/− SD, *n* = 3–4.

Because oxidative/nitrosative stress is a common IRI sequela, we evaluated nitrotyrosine as a footprint of peroxynitrite (ONOO^−^), formed from the reaction of nitric oxide and superoxide (O_2_^−^) radicals (Zielonka et al., 2010). Mitochondria are a major source of superoxide during oxidative phosphorylation (OXPHOS). When exacerbated by IRI, mitochondrial superoxide is more readily available to form peroxynitrite (Radi et al., 2002) although it can also form extramitochondrially (Radi, 2013). Peroxynitrite reacts with tyrosine residues to form nitrotyrosine regardless of its source. To assess the overall burden of peroxynitrite in kidney tissue during IRI, we examined the production of nitrotyrosine in kidney sections (Figure 4). Compared to nitrotyrosine‐blocked controls, C5^+/+^ shams kidneys showed basal nitrotyrosine (Figure 4a; black arrows). C5^+/+^ IRI increased nitrotyrosine in kidney sections (Figure 4a; black arrows), including prominent staining in necrotic areas (green asterisks). Both C5^−/−^ and AV treatment reduced nitrotyrosine after IRI (Figure 4a; black arrows). AV‐treated rats displayed focal cortical nitrotyrosine near the Bowman's capsule/glomeruli (Figure 4a), possibly reflecting drug formulation‐related stress rather than a C5aR1‐specific effect. Semi‐quantitative scores in the cortex (Figure 4b) and medulla (Figure 4c) confirmed IRI‐induced nitrotyrosine that was attenuated by C5‐C5aR1 inhibition, suggesting a role for this axis in producing reactive oxidants during IRI.

### 2.4 C5‐C5aR1: A potential regulator of mitochondrial subunit proteins

Given the reduction in nitrotyrosine facilitated by C5‐C5aR1 inhibition in kidneys post‐IRI, we sought to examine mitochondrial injury as a potential source of cellular stress. We profiled representative subunits of each mitochondrial electron transport (ETC) complex by SDS‐PAGE Western blotting using isolated mitochondrial lysates, with cytosol as control (Figure S1).

First, protein levels from C5^+/+^ and C5^−/−^ rats were compared in isolated mitochondria or cytosol derived from kidneys. Isolated renal mitochondria from C5^+/+^ sham kidneys showed robust mitochondrial subunit levels (Figure S1A,B). Compared to the sham controls, C5^−/−^ kidneys exhibited significantly reduced NDUFS3 (a complex I subunit of the Q‐module hydrogenase domain) and ATP5B (a complex V subunit of the F_1_ catalytic subunit) (Figure S1B). This was an unexpected and novel observation linking C5 to mitochondrial protein regulation. C5^+/+^ IRI significantly reduced NDUFS3, UQCRC2 (a complex III core protein involved in electron transfer), MTCO‐1 (a complex IV catalytic core protein), and ATP5B (Figure S1B) protein levels, indicating dysregulation of the ETC complex and broad mitochondrial injury. Relative to the C5^+/+^ IRI group, C5^−/−^ IRI further decreased MTCO‐1 and increased ATP5B (Figure S1B).

As expected, most mitochondrial proteins were absent from cytosol (Figure S1A,C), except SDHA, which was detected in cytosolic fractions of both C5^+/+^ and C5^−/−^ shams (Figure S1C). SDHA is nuclear‐encoded and synthesized in the cytosol; so its detection may reflect transient cytosolic pools, off‐target retention, or trafficking differences in this transgenic rat line.

To determine C5aR1‐specific effects, we considered AV‐treated rat kidneys exposed to sham or IRI surgery (Figure S1D). As in C5^−/−^ shams, AV‐treated sham rats displayed decreased mitochondrial NDUFS3 and increased SDHA compared with C5^+/+^ shams (Figure S1E). Unlike C5^−/−^ shams, AV treatment alone did not reduce ATP5B, suggesting developmental effects of lifelong C5 deficiency. Cytosolic SDHA was also detected in AV‐treated sham rats and preserved after IRI (Figure S1D,F). Taken together, these data suggest that the C5‐C5aR1 axis appears to influence mitochondrial protein dynamics, particularly ETC.

### 2.5 Effect of C5‐C5aR1 on mitochondrial complex formation

Mitochondrial ETC proteins are organized into higher order multimeric “complexes” to facilitate electron transport and ATP production. Because we noted that C5‐C5aR1 inhibition impacted the levels of several key mitochondrial ETC proteins, we sought to determine the impact of the C5‐C5aR1 axis on mitochondrial ETC organization. To achieve this, we assessed native ETC complex organization by blue native gel electrophoresis (BN‐PAGE) after mild solubilization of isolated mitochondrial membranes with lauryl maltoside (LM) (Figure S2A,B). By utilizing this strategy, the native structure of each ETC complex was visualized within Coomassie stained gels at a known molecular weight. While C5^+/+^ sham controls displayed characteristic ETC bands after Coomassie staining (Figure S2), C5^−/−^ (Figure S2A) and AV‐treated (Figure S2B) rats exhibited clear differences, particularly in the complex I region.

To better differentiate between mitochondrial complex bands after BN‐PAGE, native mitochondrial membrane proteins from BN‐PAGE were transferred for Western blotting. Immunoblotting with representative subunit antibodies (Table 1) enabled densitometric quantification normalized to total protein per lane. C5^+/+^ shams displayed robust levels of mitochondrial complexes I–V (Figure S2). Compared to the C5^+/+^ baseline level, C5^−/−^ shams displayed significantly decreased complex I (Figure S2C,D, III) (Figure S2G,H and IV) (Figure S2I,J), but increased complex II (Figure S2E,F) and V (Figure S2K,L), indicating broad remodeling of mitochondrial complexes. After C5^+/+^ IRI, complexes III (Figure S2G,H) and V (Figure S2K,L) were reduced in kidney mitochondria when compared to C5^+/+^ shams, consistent with known IRI‐mediated mitochondrial disruption (Chen et al., 2024). Notably, C5^−/−^ did not preserve mitochondrial complexes post‐IRI (Figure S2C–J).

**FIGURE 5:**
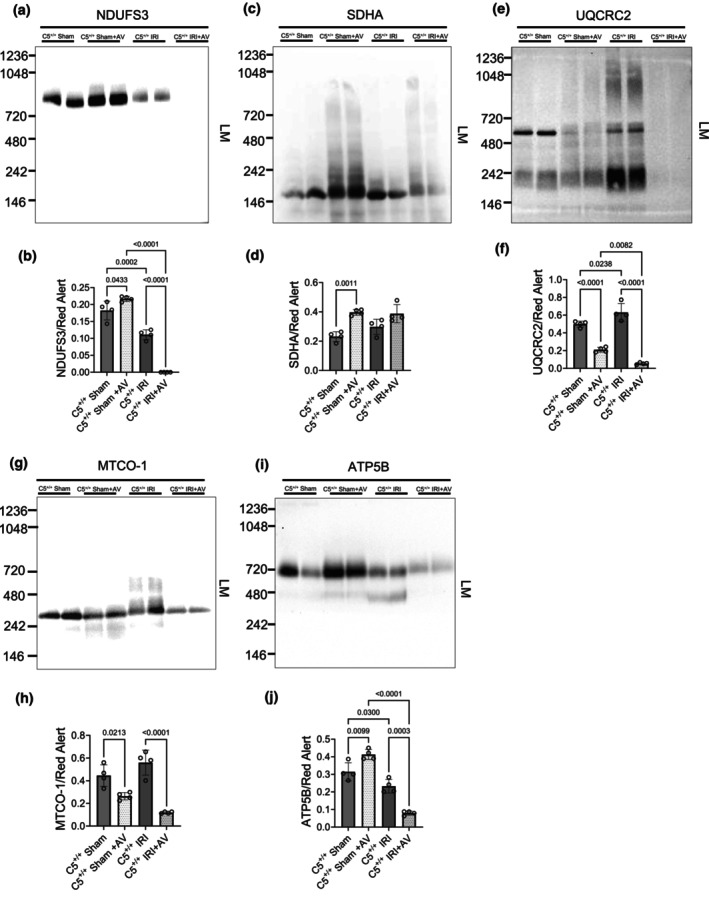
Effect of C5aR1 on the levels of renal mitochondria electron transport complexes. Renal mitochondrial membranes from male wild‐type (C5^+/+^) and AV‐treated (C5^+/+^+AV) rats were isolated and solubilized with 10% lauryl maltoside after sham or IRI surgery. 10 μg of solubilized protein per sample was resolved using BN‐PAGE for downstream Western blotting (antibodies were employed as shown in Table 1) applications. For C5^+/+^ and AV‐treated (C5^+/+^+AV) experimental group comparisons, representative Western blots display the levels of mitochondrial electron transport complexes (a) I, (c) II, (e) III, (g) IV, and (i) V, respectively. Corresponding bar graphs (b, d, f, h, j) indicate the densitometric ratio of each electron transport complex band to the total protein per gel lane. Data are shown as the mean +/− SD, *n* = 4.

In AV‐treated shams, complexes III (Figure 5e,f) and IV were decreased (Figure 5g,h), whereas complexes I (Figure 5a,b), II (Figure 5c,d), and V (Figure 5i,j) were increased. Thus, AV partially phenocopied C5 deficiency, but diverged at complex I. This potentially reflects that 24 h AV dosing was insufficient to observe differences in complex I assembly, in contrast to lifelong C5 gene deletion. Post‐IRI, complex II was decreased in C5^−/−^ but not in AV‐treated rats (Figure 5c,d), paralleling preserved SDHA protein levels.

### 2.6 Mitochondrial function and ATP levels

**FIGURE 6:**
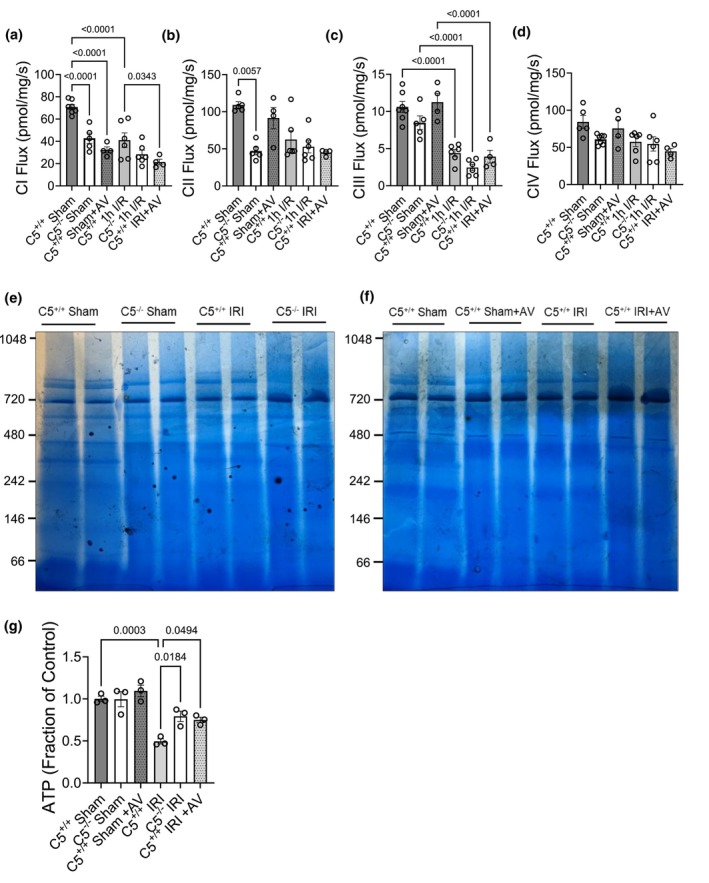
The impact of C5‐C5aR1 inhibition on renal mitochondrial function. (a–d) Fresh kidney biopsies (5–7 μg) were isolated from male wild‐type (C5^+/+^), homozygous (C5^−/−^), and AV‐treated (C5^+/+^+AV) rats after sham or IRI surgery. The biopsies were permeabilized with saponin, and the mitochondrial complex respiratory activity was analyzed using a substrate‐inhibitor titration (SIT) protocol via high resolution respirometry (HRR). Corresponding bar graphs indicate the respiration of complexes (a) I, (b) II, (c) III, and (d) IV across all experimental groups. Data are depicted as the mean +/− SD, *n* = 4–7. (e–h) To investigate the effect of C5‐C5aR1 on ATP hydrolase activity, renal mitochondrial membranes from male C5^+/+^, C5^−/−^, and C5^+/+^+AV rats were isolated and solubilized with 10% lauryl maltoside after sham or IRI surgery. 10 μg of solubilized protein per sample was resolved using BN‐PAGE for downstream in‐gel complex V activity assays. (e) For C5^+/+^ and C5^−/−^ group comparisons, a representative gel depicting the dark, brown precipitate band representing ATP hydrolytic activity is shown. (f) For C5^+/+^ and AV‐treated (C5^+/+^+AV) group comparisons, a representative gel depicting the dark precipitate band formed from ATP hydrolysis is shown. (g) Bar graph displaying the total ATP levels present in kidney extracts across the experimental groups, measured using a luciferase‐based assay. Data are shown as the mean +/− SD, *n* = 3.

The observation that the C5‐C5aR1 axis inhibition altered the organizational landscape of ETC prompted further investigation into impacts on ETC function. Fresh kidney tissue biopsies were utilized for high‐resolution respirometry (HRR) analysis using an established substrate‐inhibitor titration (SIT) protocol (Parajuli et al., 2017; Shrum et al., 2016, 2020). C5^+/+^ sham controls displayed baseline respiration in complexes I‐IV (Figure 6a–d). Notably, both C5^−/−^ and AV‐treated shams exhibited significantly decreased complex I respiration from the baseline (Figure 6a). C5^−/−^, but not AV treatment, additionally conferred a significant decrease in complex II respiration from the C5^+/+^ sham baseline (Figure 6b). This indicated that C5‐C5aR1 axis inhibition impacted ETC complex respiration in addition to complex native levels. Post‐IRI, the respiration of complexes I (Figure 6a) and III (Figure 6c) was decreased in C5^+/+^ rats compared to the C5^+/+^ sham baseline. Complex IV respiration (Figure 6d), by contrast, was not affected by C5‐C5aR1 axis inhibition or IRI.

A limitation of the HRR technique is the inability to assess the activity of complex V, the structure responsible for ATP synthesis and hydrolysis; therefore, we assessed ATP hydrolysis using an in‐gel activity assay following BN‐PAGE (Figure S3) (Jha et al., 2016). Basal ATP hydrolysis (precipitate formation) at the complex V molecular weight was evident in C5^+/+^ shams (Figure 6e,f; brown precipitates). Neither C5–C5aR1 inhibition nor IRI alone changed ATP hydrolysis from the C5^+/+^ baseline; however, both C5^−/−^ and AV markedly increased hydrolytic precipitate after IRI when compared to sham and C5^+/+^ IRI groups (Figure 6e,f), suggesting enhanced ATPase activity post‐injury.

When the respiratory activity of one or more mitochondrial complexes is altered, it is expected to have some equivocal impact on the production of ATP. Thus, to assess the effect of C5‐C5aR1 on renal ATP generation, the total level of ATP in kidney extracts was measured via a luciferase‐based assay and normalized to the ATP levels of healthy kidney controls (Figure 6g). Total renal ATP did not differ among shams (Figure 6g). IRI reduced ATP by ~50%, whereas both C5^−/−^ and AV treatment preserved ATP to near‐sham levels (Figure 6g), indicating ATP preservation by mechanisms beyond isolated ETC complex respiratory activity.

### 2.7 Role of C5‐C5aR1 in mitochondrial supercomplex organization

To reconcile reduced mitochondrial complex respiratory activity with preserved ATP, we hypothesized that C5‐C5aR1 inhibition promotes supercomplex formation, which can enhance ETC efficiency by spatially organizing complexes (Lapuente‐Brun et al., 2025). To visualize supercomplex structures, isolated mitochondrial membranes were solubilized using digitonin, a milder nonionic detergent which preserves higher‐order assemblies compared to LM. We then analyzed the digitonin solubilized membranes by BN‐PAGE followed by Coomassie staining or Western blotting (Figure S4).

Coomassie stained gels showed distinct supercomplex bands (> ~ 800 kDa) in C5^+/+^ sham controls (Figure S4A,B). This baseline banding pattern was altered in C5^−/−^ (Figure S4A) and AV‐treated (Figure S4B) shams, particularly in the supercomplex region above ~800 kDa. As expected IRI, which is known to drive mitochondrial supercomplexes disintegration (Jang et al., 2017), further disrupted these structures (Figure S4A,B). Western blots of digitonin and LM BN‐PAGE membranes (normalized to total lane protein) using antibodies against representative subunits of complex I‐V (Table 1) were evaluated side‐by‐side (Figure S4C–V). In C5^+/+^ shams, “monomeric” complexes and their supercomplexes were clearly resolved: complex I (~800 kDa) with multiple higher‐order bands (Figure S4C), complex II as a monomer (~180–200 kDa) lacking supercomplexes (Figure S4E), complex III (~600 kDa) with supercomplexes (Figure S4G), complex IV (~415 kDa) with higher‐order structures (Figure S4I), and complex V (~720 kDa) with an additional higher‐order band (Figure S4K).

Contrary to our hypothesis, the C5^−/−^ sham group displayed an overall loss of supercomplexes containing complexes I (Figure S4C), III (Figure S4G), and IV (Figure S4I), while complex V‐containing higher‐order structures were maintained (Figure S4K). C5^+/+^ IRI caused the expected supercomplex disintegration (Figure S4C,G,I,K). Strikingly, C5^−/−^ preserved baseline supercomplexes after IRI and formed additional complex I‐ (Figure S4C) and complex III‐containing supercomplexes (Figure S4G) not present at their baseline, suggesting the preservation/reorganization of some supercomplexes during injury. AV‐treated sham rats similarly showed the loss of complex I/III/IV supercomplexes (Figure S4M,Q,S) with preservation of complex V‐containing structures (Figure S4U). After IRI, AV treatment maintained its baseline supercomplex structures but, unlike C5^−/−^, did not form new assemblies (Figure S4M,Q,S,U). Overall, inhibition of the C5‐C5aR1 axis did not enhance physiological supercomplex assembly at baseline, and the supercomplex responses observed during IRI differed between the genetic and pharmacological interventions.

### 2.8 C5‐C5aR1 and the IF1 protein

**FIGURE 7:**
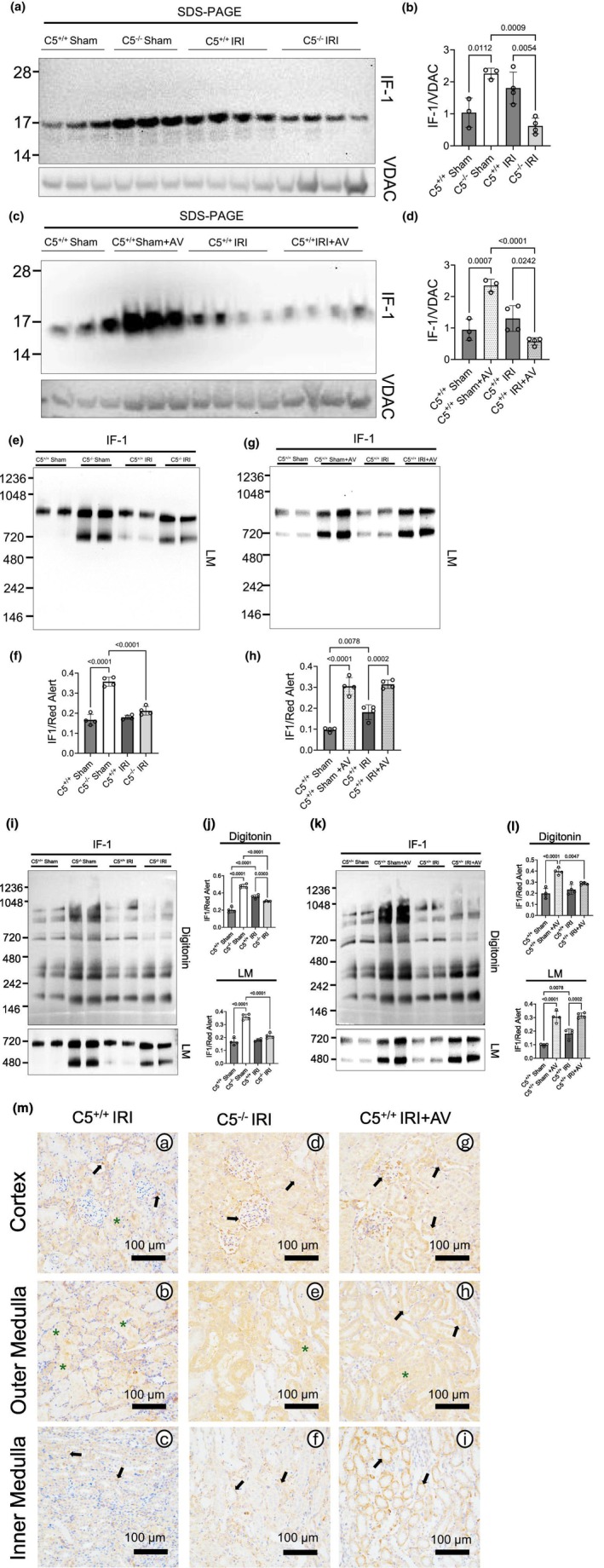
C5‐C5aR1 inhibition induces ATPase Inhibitory Factor 1 (IF1). (a–d) Renal mitochondria from male wild‐type (C5^+/+^), homozygous (C5^−/−^), and AV‐treated (C5^+/+^+AV) rats (*n* = 4 per group) were isolated and lysed with RIPA after sham or IRI surgery. 10 μg of RIPA lysate per sample was resolved through SDS‐PAGE (4%–12% gradient gel) and Western blotting was performed using antibodies as outlined in Table 1. (a, c) Representative Western blots of renal mitochondria isolated from (a) C5^+/+^ and C5^−/−^ experimental groups as well as (c) C5^+/+^ and C5^+/+^+AV experimental groups, respectively. Corresponding bar graphs depict (b, d) densitometric analysis of IF1 protein levels. Data are shown as the mean +/− SD, *n* = 3–4. (e‐h) Renal mitochondrial membranes were solubilized with 10% lauryl maltoside and visualized using BN‐PAGE. (e, g) Representative Western blots were probed with IF1 antibody for interactions with native mitochondrial membrane structures for (e) C5^+/+^ and C5^−/−^ as well as (g) C5^+/+^ and C5^+/+^+AV experimental group comparisons. Corresponding bar graphs reflect (f, h) the densitometric ratio of IF1 to the total protein per gel lane. Data are shown as the mean +/− SD, *n* = 4. (I–L) Renal mitochondrial membranes were solubilized with 10% digitonin and visualized using BN‐PAGE. (i, k) Representative Western blots were probed with IF1 antibody for interactions with supramolecular structures, or supercomplexes, for (i) C5^+/+^ and C5^−/−^ as well as (k) C5^+/+^ and C5^+/+^+AV experimental group comparisons. Corresponding bar graphs show (j, l) the densitometric ratio of IF1 to the total protein per gel lane. Data are shown as the mean +/− SD, *n* = 4. The respective representative images and densitometric ratios of mitochondrial membranes solubilized with 10% lauryl maltoside are shown for comparison. (m) Representative micrographs (*n* = 4 per group) from formalin‐fixed kidney sections after IRI, immunohistochemically stained for IF1. Black arrows indicate glomerular and tubular IF1 staining, and green asterisks indicate intratubular staining.

Because supercomplex remodeling did not explain ATP preservation, we examined ATPase Inhibitory Factor 1 (IF1), a dimeric ~16 kDa regulator that binds the catalytic head of complex V (ATP synthase) and modulates both ATP synthesis and hydrolysis (Boreikaite et al., 2019; Sgarbi et al., 2018). First, we utilized SDS‐PAGE and Western blotting to determine the impact of the C5‐C5aR1 axis on the protein level of IF1 in isolated mitochondrial lysates from rat kidneys. In C5^+/+^ sham controls, a robust baseline level of IF1 protein was observed (Figure 7a–d). However, IF1 was significantly induced in both C5^−/−^ (Figure 7a,b) and AV‐treated (Figure 7c,d) sham rats, suggesting that the C5‐C5aR1 axis suppresses IF1 at baseline within renal mitochondria.

We additionally observed the impact of IRI on the IF1 protein level in renal mitochondria. IRI did not change IF1 in C5^+/+^ kidneys (Figure 7a–d), but significantly reduced IF1 in C5^−/−^ (Figure 7a,b) and AV‐treated (Figure 7c,d) IRI groups when compared to their respective sham baselines or the C5^+/+^ IRI group (Figure 7a–d). This indicates context‐dependent regulation of IF1 during renal IRI.

Notably, prior studies have reported IF1 binding only at its canonical site on complex V (Gu et al., 2019). To assess IF1 interactions with mitochondrial assemblies, including complex V, under baseline conditions and during C5‐C5aR1 inhibition, we probed LM BN‐PAGE membranes for IF1 (Figure 7e–h). In C5^+/+^ sham rats, IF1 signal was predominantly associated with higher‐order structures >800 kDa rather than exclusively with the monomeric complex V band (~ 720 kDa) (Figure 7e,g), consistent with IF1 bridging complex V dimers. In both C5^−/−^ and AV‐treated shams, IF1 showed increased association with the monomeric complex V band, and this pattern was maintained post‐IRI (Figure 7e–h).

Digitonin BN‐PAGE Western blotting further revealed multiple IF‐1 associated bands both within and below the >800 kDa region in C5^+/+^ sham controls (Figure 7i,k), indicating interactions beyond the canonical complex V site. These interactions persisted across conditions regardless of C5‐C5aR1 inhibition or IRI (Figure 7i–l). Together, these observations prompted further exploration into IF1's localization and its potential role(s) in renal mitochondria in the context of C5 proficiency or deficiency.

### 2.9 Prevalence of IF1 in kidney tissue

To investigate IF1's localization in the kidneys, formalin‐fixed and paraffin‐embedded kidney sections were immunohistochemically stained for the IF1 protein (Figure S5). Our results showed robust IF1 staining throughout the kidney tissue in C5^+/+^ sham controls (Figure S5a–c), with enrichment in glomeruli (Figure S5a; black arrows) and proximal tubules (Figure S5b; black arrows) and relatively low signal within the inner medullary region (Figure S5c). Both C5^−/−^ (Figure S5d–f) and AV‐treated (Figure S5g–i) shams displayed increased IF1 across the kidney tissue, including enhanced staining in distal nephron of the inner medulla (Figure S5f,l; black arrows). This was notable, given the inner medulla's hypoxic milieu and susceptibility to IRI (Layton, 2016).

After IRI (Figure 7m), IF1 remained predominantly localized to glomeruli and proximal tubules (Figure 7m; black arrows) with extracellular leakage in necrotic regions (green asterisks). C5^+/+^ IRI also revealed inner medullary IF1 not seen at the sham baseline (Figure 7m; black arrows). In C5^−/−^ and AV‐treated IRI groups, IF1 staining persisted in glomeruli and proximal tubules (Figure 7m; black arrows) with some inner medullary signal (Figure 7m; black arrows), but IF1 staining was noticeably blunted compared to their respective C5^−/−^ and AV‐treated sham controls. Thus, C5‐C5aR1 inhibition induced IF1 and altered its compartmental localization.

### 2.10 IF1 induction and localization in proximal tubular cells

To differentiate intracellular from circulating complement effects, we manipulated C5 (genetically with siRNA) or C5aR1 (pharmacologically with AV) in normal rat kidney proximal tubular (NRK) cells (Figure S6). Transient C5 siRNA knockdown reduced C5 protein levels when compared to untreated and scrambled controls (Figure S6A,B) and significantly induced IF1 (Figure S6C) mirroring C5^−/−^ kidneys. Tangentially, we sought to validate if IF1 induction in NRK cells was a C5aR1‐specific effect. NRK cells were treated with increasing cumulative doses of AV or equivalent volumes of vehicle (VEH) over 48 h. SDS‐PAGE Western blotting and subsequent densitometry analysis confirmed IF1 protein induction after C5aR1 inhibition via AV treatment (Figure S6D,E) without affecting cell viability (Figure S6F). A cumulative 100 μM AV over 48 h was used for the subsequent in vitro studies.

**FIGURE 8:**
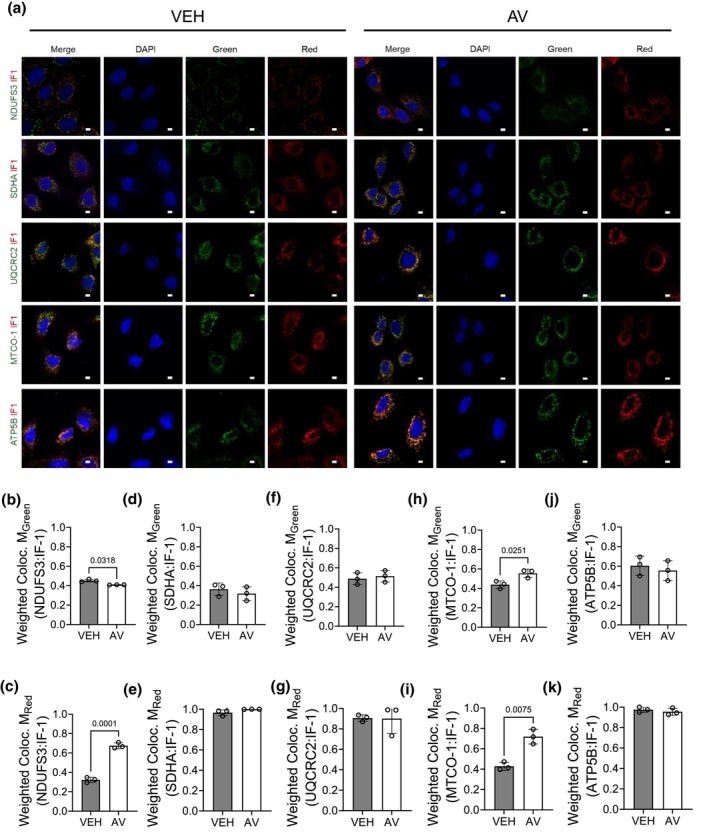
Avacopan promotes IF1 colocalization with mitochondrial complexes I and IV in proximal tubular cells. NRK cells were exposed to AV (100 μM cumulative dose) or an equivalent volume of VEH control for a treatment period of 48 h, then were immunocytochemically labeled with IF1 (red) or other mitochondrial protein antibodies (green) as outlined in Table 1. DAPI was used to label cell nuclei. (a) Representative micrographs reflect the immunofluorescent signal in NRK cells, imaged in z‐stacks using an LSM 880 confocal microscope plus Airyscan (100× magnification). Scale bar = 100 pixels. Corresponding bar graphs (b–k) display the quantification of fractional colocalization, as represented by weighted Mander's Colocalization Coefficients. M_Green_ = the fractional colocalization of the green fluorescent signal overlapping with red fluorescent signal, and M_Red_ = the fractional colocalization of the red fluorescent signal (IF1) overlapping with green fluorescent signal. Data are shown as the mean +/− SD, *n* = 3 individual experiments.

To assess subcellular localization, we treated NRK cells with vehicle (VEH) or AV and performed confocal z‐stacks and colocalization (Manders' coefficient) of IF1 (red; fraction reported as M_Red_) with representative markers for ETC complexes (green; fraction reported as M_Green_) (Manders et al., 1993) (Figure 8a). In preliminary experiments, we confirmed that IF1 colocalized primarily with the inner mitochondrial membrane rather than other mitochondrial compartments (Figure S7). As expected, M_Green_ values were moderate across groups (Figure 8b,d,f,h,j). In VEH‐treated cells, IF1 showed high fractional colocalization (M_Red_ = ~1.0) with complexes II, III, and V (Figure 8e,g,k). AV significantly increased IF1 colocalization with complexes I and IV (Figure 8c,i), corroborating BN‐PAGE evidence that C5aR1 inhibition alters IF1's interactions within the inner mitochondrial membrane.

### 2.11 C5‐C5aR1 inhibition promotes IF1's regulatory function within mitochondria

**FIGURE 9:**
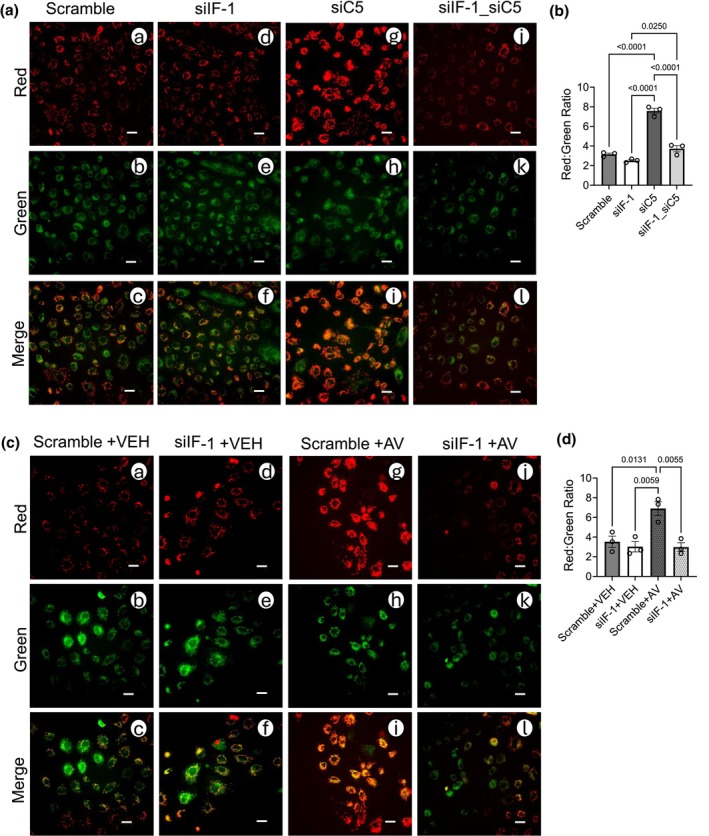
C5‐C5aR1 inhibition promotes IF1‐dependent mitochondrial membrane hyperpolarization in proximal tubular cells. (a, b) NRK cells (*n* = 3 individual experiments) were transiently transfected with siRNA targeted to IF1 (siIF1), C5 (siC5), or a scrambled control. 48 h posttransfection, cells were exposed to JC‐1 dye to assess the mitochondrial membrane potential. (a) Representative micrographs of live NRK cells were taken with a Nikon Eclipse E800 microscope at 60× magnification in Nikon Elements software. Red = fluorescent signal corresponding to J‐aggregates. Green = fluorescent signal corresponding to JC‐1 dye monomers. Scale bar = 100 pixels. (b) Bar graph of the red J‐aggregate: Green monomer ratio, as quantified using a BioTek microplate reader. Data are shown as the mean+/− SD, *n* = 3. (c, d) NRK cells (*n* = 3 individual experiments) were transiently transfected with siRNA targeted to IF1 (siIF1) or a scrambled control. Subsequently, cells were treated with AV (100 μM cumulative dose) or VEH over a 48 h period posttransfection. After the treatment period, cells were exposed to JC‐1 dye. (c) Representative micrographs of live NRK cells were imaged using the conditions described in (a). (d) Bar graph of the red J‐aggregate: Green monomer ratio, as described in (b). Data are shown as the mean +/− SD, *n* = 3.

To further investigate the novel functional relationship between the C5‐C5aR1 axis and IF1 within mitochondria, we genetically (siRNA) modulated C5/IF1 or pharmacologically inhibited C5aR1 in NRK cells and assessed the mitochondrial membrane potential using JC‐1 dye (Figure 9). IF1 regulates the mitochondrial membrane potential by modulating complex V‐dependent ATP synthesis or hydrolysis and therefore, proton flux (Sánchez‐Cenizo et al., 2010). In both Scramble siRNA (Figure 9a a‐c) and VEH‐treated (Figure 9c a‐c) controls, the green JC‐1 monomer is localized to mitochondria, and a sufficiently negative membrane potential promoted the formation of red JC‐1 aggregates, suggesting a robust mitochondrial membrane potential. While the knockdown of IF1 did not significantly impact the membrane potential (Figure 9b,d; quantified as the red aggregate: green monomer ratio), both C5 knockdown (Figure 9a) and AV treatment (Figure 9c) hyperpolarized the membrane potential in NRK cells. This hyperpolarization effect was lost after cumulative knockdown with IF1 (Figure 9a and Figure 9c), indicating IF1 dependence.

**FIGURE 10:**
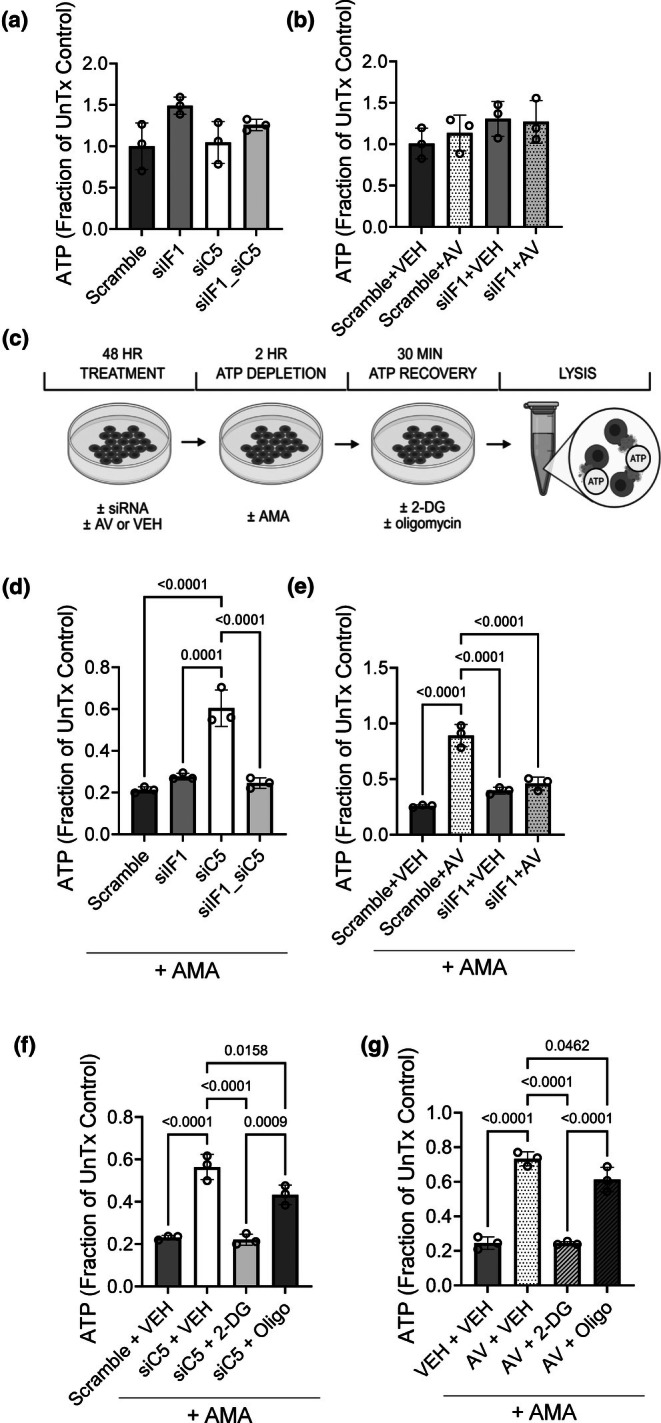
C5‐C5aR1 inhibition improves IF1‐dependent ATP recovery after AMA‐mediated depletion in proximal tubular cells. (a, b) NRK cells (*n* = 3 individual experiments) were transiently transfected with siRNA targeted to IF1 (siIF1), C5 (siC5), or a scrambled control. To assess C5aR1‐specific effects, cells were treated with AV (100 μM cumulative dose) or VEH over a 48 h period posttransfection. After the treatment period, cells were lysed and the total ATP levels were quantified using a luciferase‐based assay. Bar graphs depict the total ATP, normalized as a fraction of the ATP level in untreated (UnTx) control NRK cells. Data are shown as the mean +/− SD, *n* = 3. After this baseline quantification of ATP levels, NRK cells were exposed to AMA for ATP depletion as depicted by the schematic (c). (d, e) Bar graphs reflecting the total ATP levels in NRK cell treatment groups described in (a, b), after 2 h of AMA exposure and 30 min of ATP recovery. Data are shown as the mean +/− SD, *n* = 3. (f, g) NRK cells (*n* = 3 individual experiments) were transiently transfected with siRNA targeted to C5 (siC5) or treated with AV (100 μM cumulative dose) to distinguish C5aR1‐specific effects. After a 48 h treatment period, cells were exposed to AMA for 2 h of ATP depletion. During a subsequent 30 min period of ATP recovery, NRK cells were treated with 2‐DG, Oligo, or VEH as shown in the schematic (c). Bar graphs show the total ATP levels post‐ATP recovery as a fraction of UnTx controls. Data are displayed as the mean +/− SD, *n* = 3.

To determine the ultimate impact on cellular ATP levels, NRK cells were considered for C5/C5aR1 and/or IF1 modulation, followed by quantification of ATP levels in cell extracts (Figure 10a,b). Scramble (Figure 10a) and VEH‐treated (Figure 10b) controls displayed a total quantity of ATP comparable to untreated (UnTx) NRK cell controls. In healthy NRK cells, the total ATP was unchanged by IF1 knockdown, C5 knockdown, or AV treatment (Figure 10a,b), consistent with our in vivo sham results. To model IRI‐like ATP loss, we used antimycin A (AMA; complex III inhibitor) to deplete ATP for 2 h, followed by 30 min of ATP recovery (Figure 10c). AMA reduced ATP to <50% of the baseline levels in controls (Figure 10d,e). Both C5 siRNA and AV significantly enhanced ATP recovery (Figure 10d,e). This reflected our in vivo results, which showed that C5‐C5aR1 axis inhibition preserved the ATP levels in kidneys after IRI. However, when IF1 knockdown was paired with C5 knockdown or AV treatment, ATP recovery after AMA‐mediated depletion was significantly blunted (Figure 10d,e), suggesting IF1‐dependent rescue. To delineate the processes contributing to ATP recovery after C5‐C5aR1 inhibition, NRK cells were treated with inhibitors for glycolysis (2‐Deoxy‐D‐glucose, or 2‐DG) and complex V (oligomycin, or Oligo) during the ATP recovery period (Figure 10f,g). Glycolysis and the ATP synthase were selected as targets due to their known regulation by IF1 and ability to generate ATP (Sánchez‐Cenizo et al., 2010; Zhou et al., 2022). Inhibiting glycolysis using 2‐DG fully abrogated recovery, while Oligo partially reduced it (Figure 10f,g). Taken together, our results demonstrate that the inhibition of C5‐C5aR1 axis induces mitochondrial IF1 and promotes cellular ATP recovery primarily through glycolysis.

## 3 DISCUSSION

We identify a previously unrecognized link between the C5‐C5aR1 axis and IF1 in renal mitochondrial physiology and IRI. While prior studies in T‐cells (Liszewski et al., 2013; Sena et al., 2013) establish a link between C5‐C5aR1 and OXPHOS, to the best of our knowledge there are no previous studies in any organ systems linking this axis (or the pharmacological agent Avacopan) to IF1. Inhibiting the C5‐C5aR1 axis by genetic C5 deletion (C5^−/−^) or pharmacologic C5aR1 blockade with Avacopan (Tavneospro, 2023) preserved kidney function and tissue morphology. C5‐C5aR1 inhibition also reduced injury biomarker deposition, attenuated apoptosis/necrosis, and decreased nitrotyrosine formation after IRI. Building on these protective effects, we investigated mitochondrial mechanisms linking the C5‐C5aR1 axis to bioenergetics regulation.

The key findings of this study reveal, for the first time, that the C5‐C5aR1 axis is complexly intertwined with the mitochondrial ETC. C5‐C5aR1 axis inhibition altered the abundance of several mitochondrial ETC subunits and remodeled native ETC complex levels in healthy kidneys, indicating basal effects on mitochondrial physiology. Using HRR, a cutting‐edge technique (Djafarzadeh & Jakob, 2017), we found that both C5^−/−^ and C5aR1 inhibition (AV) reduced baseline complex I respiration, yet ATP levels were preserved in shams and protected after IRI. Supercomplex analysis showed no enhancement of baseline assembly; instead, both C5^−/−^ and AV reduced supercomplexes containing complexes I, III, and IV. Intriguingly, C5^−/−^ kidneys preserved and even formed additional supercomplexes after IRI, whereas AV maintained (but did not expand) baseline assemblies, suggesting context‐ and timing‐specific remodeling (lifelong C5 loss versus acute pharmacological inhibition).

Because ATP preservation could not be explained by supercomplex remodeling alone, we examined IF1, a canonical regulator of the ATP synthase (Boreikaite et al., 2019; García et al., 2006; Gu et al., 2019). Beyond its traditional role, IF1 upregulation has been linked to aerobic glycolysis in cancer cells (Sgarbi et al., 2018) and murine cardiovascular models (Zhou et al., 2022). Thus, IF1 can influence ATP levels via multiple potential avenues. C5‐C5aR1 axis inhibition robustly induced mitochondrial IF1, shifted its association toward monomeric complex V on LM BN‐PAGE, and revealed its associations with multiple higher‐order assemblies on digitonin BN‐PAGE. To our knowledge, this is the first report linking IF1 to the C5‐C5aR1 axis in renal mitochondria. In proximal tubular cells, C5 knockdown or AV similarly increased IF1 abundance, enhanced its colocalization with complexes I and IV, hyperpolarized the mitochondrial membrane potential, and promoted ATP recovery after chemical depletion. In kidney sections, C5^−/−^ or AV treatment increased IF1 throughout the kidney but particularly in the medulla, an area susceptible to necrosis and other ischemic sequelae (Layton, 2016). This was a critical finding, as robust IF1 induction in the medulla coordinates with attenuated renal tissue injury observed in C5^−/−^ and AV‐treated rats post‐IRI, suggesting IF1 exerts protective effects in the medullary region. Importantly, ATP recovery was IF1‐dependent and driven predominantly by glycolysis, as 2‐DG (a glycolysis inhibitor) abolished recovery while oligomycin (a complex V inhibitor) only partially reduced it. Thus, under physiological conditions, the intracellular C5‐C5aR1 axis suppresses IF1 and limits its association with ETC complexes. This constrains IF1‐facilitated glycolysis and ATP maintenance, potentially sensitizing renal cells to ATP loss during injury.

Our findings align with previous reports that C5aR1 inhibition preserves renal function and mitigates IRI (Arias‐Cabrales et al., 2021; Arumugam et al., 2003; Peng et al., 2019). Given complement's broad physiological roles, therapeutically targeting upstream components risks widespread disruption, whereas focusing downstream component is advantageous. Clinically, C5 (part of the terminal complement cascade) is an attractive therapeutic target to ameliorate complement‐driven pathologies (Kolev et al., 2024). AV, used throughout this study, is a clinically relevant and reversible competitive C5aR1 antagonist that spares C5b (Garg & Frishman, 2023), thereby preserving a key part of the terminal cascade. Using both C5^−/−^ and AV enabled delineation of C5aR1‐specific renal and mitochondrial effects. As some discrepancies were observed between C5^−/−^ and AV treatment, future studies should address potential contributions of C5b/MAC and other complement proteins in mitochondrial energetics.

In this study, we identified broad C5‐mediated regulation of mitochondrial subunits and ETC complexes, although not all effects were C5aR1‐specific. At the subunit level, C5‐C5aR1 inhibition reduced the abundance of NDUFS3 (of complex I) and ATP5B (of complex V). NDUFS3, a conserved subunit within the hydrogenase domain of complex I (Samanta et al., 2021), is linked to mitochondrial dysfunction and increased aerobic glycolysis when reduced (Suhane et al., 2013), consistent with the impaired complex I respiration observed in fresh C5^−/−^ or AV‐treated kidney biopsies. ATP5B, a key catalytic subunit of ATP synthase (Brüggemann et al., 2017), is associated with reduced ATP production when deficient (Nasca et al., 2023). Total ATP levels remained unchanged after C5‐C5aR1 inhibition, suggesting either insufficient ATP5B loss to impact synthesis or compensatory ATP production through alternative pathways. Contrary to our hypothesis, supercomplexes did not explain ATP preservation. Instead, IF1 emerged as a plausible compensatory mechanism. Both C5^−/−^ and C5aR1 inhibition markedly induced IF1 and broadened its interaction landscape beyond the canonical complex V binding, identifying a C5aR1‐specific effect on IF1 regulation in renal mitochondria.

Because the source of C5‐C5aR1 signal is pivotal (Farrar et al., 2006; Lalli et al., 2008; Pratt et al., 2002), we considered intracellular complement (the ‘complosome’), including C5aR1 on the outer mitochondrial membrane (Ishii & Rohrer, 2023; Niyonzima et al., 2021). Prior studies in immune and epithelial cells link C5a‐C5aR1 signaling to ROS generation and mitochondria‐dependent apoptosis (Silva et al., 2023; Arbore et al., 2016; Ishii & Rohrer, 2023; Tsai et al., 2019). Similarly, our group recently demonstrated that C5 gene deletion blunted IRI‐mediated caspase‐3 activation (McGraw et al., 2025). Here, we validated the novel relationship between C5‐C5aR1 and IF1 in proximal tubular cells, implicating intracellular complement as a regulator of IF1. Confocal analyses revealed that C5aR1 inhibition enhanced IF1 colocalization with complexes I and IV while maintaining associations with complexes II, III, and V. Collectively, these findings indicate that intracellular C5‐C5aR1 controls IF1 abundance and localization within renal mitochondria.

Functional C5‐C5aR1 inhibition hyperpolarized the mitochondrial membrane potential in an IF1‐dependent manner, consistent with prior reports that IF1 limits proton backflow (Sánchez‐Cenizo et al., 2010). Under antimycin A‐induced ATP‐depletion, C5‐C5aR1 inhibition preserved total ATP in an IF1‐dependent fashion; glycolytic inhibition fully abolished ATP rescue, indicating an IF1‐driven glycolytic metabolic shift (Warburg‐like phenotype) in proximal tubular cells that can preserve ATP production during stress such as IRI.

In summary, we provide a comprehensive characterization of the role of the C5‐C5aR1 axis in renal mitochondria and identify a novel regulatory relationship with IF1. Targeting C5aR1 reduced nitrotyrosine formation, mitigated tubular injury, and ameliorated IRI. Although C5‐C5aR1 inhibition impaired complex I function and reduced supercomplex formation at baseline, ATP levels were preserved during IRI. This led to the discovery that C5‐C5aR1 modulates IF1 expression and intra‐mitochondrial localization, producing functional consequences, including mitochondrial membrane hyperpolarization and ATP recovery, primarily through glycolysis. These findings reveal a unique mechanism by which C5‐C5aR1 inhibition promotes metabolic adaptation in renal cells and position the C5‐C5aR1‐IF1 axis as a promising therapeutic target for renal IRI.

### 3.1 Limitations

While our study identified a novel relationship between the C5‐C5aR1 axis and IF1, limitations arose which pave the way for future studies to investigate this complex mechanism further. A key limitation included the difficulties of modifying IF1 in vivo, and one reason for this was the lack of IF1‐targeted inhibitors. Peptides designed to displace IF1 from its canonical binding site on the ATP synthase have been developed (Grandi et al., 2024) but these inhibitors do not account for IF1's interactions with other proteins, including the remaining ETC complexes or glycolysis mediators. Furthermore, although our study addresses the abundance of IF1 along with its various interactions with the mitochondrial membrane, IF1 posttranslational modifications such as phosphorylation (Cuezva & Domínguez‐Zorita, 2024) play a role in IF1's ability to form active dimers. Future studies should assess IF1's phosphorylation status as mediated by protein kinase A (PKA) (García‐Bermúdez et al., 2015) during renal IRI. Another limitation involved the challenges associated with the varied cellular localization of C5aR1. Our ability to assess the contributions of intracellular C5aR1 was limited to in vitro studies, but either cell surface C5aR1 or mitochondrial C5aR1 may be engaged by AV. Technologies for targeting mitochondrial C5aR1 while excluding cell surface C5aR1 have yet to be developed, although distinguishing the two may prove valuable in understanding the role of the C5‐C5aR1 axis in renal mitochondria. Finally, in the above studies we have chosen to utilize male rodents as men carry a 63% higher incidence of severe kidney disease compared to women (Kidney Disease Statistics for the United States—NIDDK, 2026), but future studies should be conducted to identify any existing sex‐based differences.

### 3.2 Implications and future directions

Our study provided a mechanistic insight that C5‐C5aR1 shapes mitochondrial protein composition, complex organization, and IF1‐mediated regulation. Similarly, these findings implicate a therapeutic opportunity utilizing C5aR1 antagonism to promote IF1‐dependent ATP preservation via glycolysis during IRI. We predict a translational relevance in using Avacopan, an FDA‐approved agent, to achieve mitochondrial and metabolic benefits consistent with renal protection in the setting of IRI. Future studies should define upstream signaling linking C5aR1 to mitochondrial IF1 (e.g., kinases/phosphor‐IF1 states), resolve IF1‐associated assemblies by native mass spectrometry/complex profiling, and test whether timed C5aR1 inhibition can optimize IF1‐driven metabolic adaptation in clinically relevant IRI models.

## 4 METHODS

### 4.1 Generation of the C5 ^−/−^ rat model

The C5^−/−^ rat model used in these studies was generated by the University of Michigan Transgenic Core as previously described (McGraw et al., 2025). Briefly, fertilized Lewis rat eggs were microinjected with CRISPR/Cas9 reagents to create double‐strand breaks in C5‐201 exon 3 (chromosome 3). Base pair removal was achieved via nonhomologous end‐joining (Filipiak & Saunders, 2006). Genomic DNA was extracted from tail snip biopsies using the DNEasy DNA kit (Qiagen, #69504) and the presence of deletion products was confirmed by the Core. Heterozygous (G1) pups were shipped to the University of Arkansas for Medical Sciences and maintained in a colony.

### 4.2 Animal care and Colony maintenance

Heterozygous (C5^+/−^) male and female Lewis rats were housed in monogamous breeding pairs in animal facilities at the University of Arkansas for Medical Sciences. Breeding and housing procedures were carried out as previously described (McGraw et al., 2025). Briefly, the rats were maintained on a 12 h day/night cycle at physiological temperatures, and were fed standard chow (Lab Diet, #3002906–704) and given water ad libitum. All animal use protocols were performed as approved by the Institutional Animal Care and Use Committee (IACUC) in accordance with the National Institutes of Health (NIH) guidelines.

### 4.3 Genotype analysis

For genotyping, genomic DNA was extracted from the tail biopsies of 3–4 week old rats using the Phire Direct PCR Master Mix kit (Thermofisher Scientific, #F170S). C5 gene deletion was confirmed via PCR by using a C5 primer (Forward: 5′‐GCTTTTATTCCACCCAGGAT‐3′; Reverse: 5′‐TCCCCTTTGTGTTTGTAGGA‐3′) and observing a 334 bp PCR product. Genotyping PCR was performed as previously described (McGraw et al., 2025).

### 4.4 Animal surgery

#### 4.4.1 Ischemia–Reperfusion Injury (IRI) Procedure

Male Lewis rats (8–10 weeks old) were exposed to a surgical model of renal ischemia–reperfusion injury (IRI) as performed by our group in previous studies (McGraw et al., 2025). Male rats were utilized to reduce the overall number of animals needed for the study, as no major differences in IRI‐mediated renal injury were observed between male and female rats in our group's previous characterization of the C5^−/−^ model (McGraw et al., 2025). Furthermore, males have a significantly greater risk for renal IRI compared to females (Kidney Disease Statistics for the United States—NIDDK, 2026). Briefly, anesthetized rats were dosed with 0.1 mg/kg buprenorphine HCl administered subcutaneously (s.c.) for pain management and maintained on isoflurane anesthesia (5% induction and 2% maintenance) during all surgical procedures. Avacopan (AV; MedChemExpress LLC, #HY‐17627) was administered intraperitoneally (i.p.) at 30 mg/kg to relevant experimental groups 1 h prior to the onset of ischemia. An incision was made from the xyphoid process to the symphysis pubis region to access the interior abdomen, and the renal pedicles were clamped for a duration of 1 h to induce ischemia. After the ischemic period, the right kidney was removed and stored for use in future studies. Clamps were removed and blood flow to the left kidney was restored (reperfusion). Incision closure was carried out by suturing the muscular layer and stapling the skin over the incision site. Rats were returned to the animal facility for a 1‐day recovery period, after which the animals were re‐anesthetized for organ and blood collection. Animals were then euthanized via exsanguination as approved by IACUC.

#### 4.4.2 Sham Procedure

Sham surgeries were performed similarly to the IRI procedure, except pedicle clamping was omitted. Briefly, incisions were made in the abdomens of anesthetized rats, and the kidneys were exposed. The right kidney was surgically removed and processed as a nonsurgical “healthy” control, utilized within these studies for normalization of *ATP assay* results. Incisions were closed as in the IRI procedure, and rats were returned to the animal facilities for the 1‐day recovery period. After recovery, rats were re‐anesthetized for collection of the left kidney (sham control).

#### 4.4.3 Sample Collection

Kidneys and blood were collected from anesthetized rats immediately prior to exsanguination. Fresh whole blood was immediately processed for hematology analysis. Kidneys were dissected in a bath of sterile saline solution, on ice, for use in downstream applications. Fresh kidney biopsies (5–7 μg) were immediately processed for high‐resolution respirometry (HRR). A portion of each kidney was sectioned, formalin‐fixed, and paraffinized for histology and immunohistochemistry analysis. Kidney sections to be used in SDS‐PAGE Western blotting and ATP assays were flash‐frozen in liquid nitrogen and made into tissue powder for long‐term storage at −80°C. Renal mitochondria were isolated in sucrose‐containing buffer using an established differential centrifugation protocol (Munusamy et al., 2009; Saba et al., 2007). Isolated mitochondria and cytosolic fractions were aliquoted and flash‐frozen in liquid nitrogen for long‐term storage at −80°C. Frozen mitochondria were not thawed more than once for downstream applications such as blue‐native PAGE.

### 4.5 Hematology

Fresh whole blood was analyzed for renal function parameters using the VetScan 1 i‐STAT system (Abaxis, #04P75‐03) and CHEM8+ cartridges (Abaxis, #09P31‐26).

### 4.6 Histology and immunohistochemistry

Formalin‐fixed kidney tissue was paraffinized in blocks, and 4–5 μM thick cross‐sections were mounted on glass slides (Fisher Scientific, #12–544‐3). Cross‐sections were then deparaffinized using xylene and graded ethanol washes as previously described (McGraw et al., 2025). Deparaffinized sections were either processed for Periodic Acid‐Schiff (PAS) staining or, for immunohistochemistry, were heated in a sodium citrate buffer (pH = 6.0) for antigen retrieval. Quenching was carried out with BLOXALL™ Endogenous Peroxidase and Alkaline Phosphatase Blocking Solution (Vector, #SP‐600). After quenching, sections were blocked with 2.5% normal goat serum and incubated in primary antibodies as indicated in Table 1. ImmPRESS™ Reagent Anti‐Rabbit IgG (Vector, #MP‐7451) or Anti‐Mouse IgG (Vector, #MP‐7423) was used in conjunction with ImmPACT™ DAB Peroxidase Substrate (Vector, #SK‐4105) to detect immunoreactivity, as appropriate. Sections were counterstained with Mayer's Hematoxylin (Electron Microscopy Science, #26043) and bluing was performed using 0.125% ammonia hydroxide. Slides were then dehydrated and mounted with Cytoseal‐60 (Electron Microscopy Science, #18006). Bright‐field imaging was carried out using a Nikon Eclipse E800 microscope in Nikon Elements software. For histological and immunohistochemical analysis, at least 2 renal sections were examined per rat, and 10 randomized/blindly labeled 40X fields were examined (representing cortex and medulla) per section. A blinded and licensed pathologist performed the histopathological evaluation and acute tubular necrosis (ATN) scoring as in our prior publication (McGraw et al., 2025). An established semi‐quantitative scoring system was employed to assess renal injury biomarker and nitrotyrosine deposition (Sharma et al., 2025).

**TABLE 1:** Antibodies.

Antibody	Description	Source	Application	Dilution	Catalog No. #
C5	Complement protein 5	Proteintech	WB	1:1000	22,492‐1‐AP
NDUFS3	Mitochondrial complex I subunit	Abcam	WB, ICC	1:1000 WB 1:100 ICC	ab110246
SDHA	Mitochondrial complex II subunit	Abcam	WB, ICC	1:1000 WB 1:100 ICC	ab14715
UQCRC2	Mitochondrial complex III subunit	Abcam	WB, ICC	1:1000 WB 1:100 ICC	ab14745
MTCO‐1	Mitochondrial complex IV subunit	Abcam	WB, ICC	1:1000 WB 1:100 ICC	ab14705
ATP5B	Mitochondrial complex V subunit	Invitrogen	WB, ICC	1:1000 WB 1:100 ICC	A‐21351
IF1	Mitochondrial complex V inhibitory factor	Cell Signaling Technologies	WB, ICC, IHC	1:1000 WB 1:100 ICC 1:200 IHC	8528S
VDAC	Outer mitochondrial membrane channel	Abcam	WB, ICC	1:1000 WB 1:100 ICC	ab14734
Hsc70	Heat shock protein; chaperone	Invitrogen	WB	1:1000	MA3‐014
Β‐Actin	Cytoskeletal protein	Sigma	WB	1:1000	A5441
MnSOD	Mitochondrial matrix superoxide scavenger	Invitrogen	ICC	1:100	MA5‐31514
KIM‐1	Kidney injury biomarker	LSBio	IHC	1:2000	LS‐C312791
NGAL	Kidney injury biomarker	LSBio	IHC	1:2000	LS‐C37211
Peroxidase Goat Anti‐Mouse IgG	Secondary antibody	Jackson ImmunoResearch	WB	1:30,000	115–035‐166
Peroxidase Goat Anti‐Rabbit IgG	Secondary antibody	Jackson ImmunoResearch	WB	1:30,000	111–035‐144
Alexa Fluor 488 Goat Anti‐Mouse IgG	Secondary antibody	Invitrogen	ICC	1:10,000	A11029
Alexa Fluor 594 Goat Anti‐Rabbit IgG	Secondary antibody	Invitrogen	ICC	1:10,000	A11037

#### 4.6.1 Nitrotyrosine Staining

Immunohistochemical analysis of nitrotyrosine was performed as previously described (Parajuli et al., 2011) with an anti‐nitrotyrosine antibody as indicated in Table 1. Nitrotyrosine antibody specificity was confirmed by blocking with 10 mM 3‐nitrotyrosine (Patil et al., 2014).

### 4.7 TUNEL assay

Formalin‐fixed and paraffin‐embedded kidneys were sectioned and mounted on glass slides. Tubular necrosis and cellular apoptosis were visualized using the in‐situ terminal transferase‐mediated dUTP nick‐end labeling (TUNEL) method according to the manufacturer's protocol (TACS TdT Kit, R&D Systems, #4810‐30‐K) (TACS 2 TdT‐DAB, 2026). Necrotic tubules and TUNEL‐positive nuclei were semi‐quantitatively evaluated by counting the number of tubules/nuclei in 10 randomized and blind‐labeled 40x fields (representing cortex and medulla) per kidney section. Per rat, at least 2 separate kidney sections were examined.

### 4.8 High resolution respirometry

Fresh kidney biopsies (5–7 μg) containing cortex and medulla were permeabilized with 100 μg/mL saponin in MiRO5 (60 mM K‐lactobionate, 0.5 mM EDTA, 3 mM MgCl_2_, 20 mM taurine, 10 mM KH_2_PO_4_, 20 mM HEPES, 110 mM sucrose, and 1 g/L BSA) buffer and the mitochondrial respiratory complex activity was assessed using high‐resolution respirometry (HRR) using an established substrate‐inhibitor titration (SIT) protocol (Parajuli et al., 2017; Shrum et al., 2016, 2020).

Briefly, permeabilized biopsies were introduced into the Oxygraph‐2 k chamber (Oroboros Instruments, Innsbruck, Austria) and a stopper was inserted to close the chamber. Mitochondrial respiration was initiated by adding 2 mM malate and 10 mM glutamate (Complex I substrates; Sigma, #M0875 and #1446600 respectively) into each chamber through the stopper using a Hamilton syringe. 2.5 mM ADP was added to achieve maximal oxidative phosphorylation‐dependent respiration. Next, 100 μM rotenone (Complex I inhibitor; Sigma, #R8875) was added into the chamber to reduce substrate‐induced respiration. Subsequently, 10 mM succinate (Sigma, #S‐2378) was added to initiate Complex II and III respiration. The substrate‐induced respiration was inhibited by adding 2 mM malonate (Complex II inhibitor; Sigma, #M4795) followed by 10 μM antimycin A (Complex III inhibitor; Sigma, #A8674). To stimulate Complex IV respiration, 5 μM tetramethyl‐p‐phenylenediamine (TMPD; Millipore, #T3134; stabilized with ascorbate) was added to the chamber followed by the Complex IV inhibitor, 250 mM azide. Analysis of mitochondrial respiration for each complex was carried out using Oroboros DATLAB 4.2 software.

### 4.9 ATP assay

The total ATP level in kidney/cell lysates was detected using a luciferase‐based ATP assay according to the kit (ATP Determination Kit, Invitrogen, #A22066) manufacturer's protocol (ATP Determination Kit 1 Kit | Buy Online | Invitrogen™, 2026). Quantification was carried out using a BioTek microplate reader (Agilent Technologies, Santa Clara, California) for 3 experimental replicates per rat and/or cell experiment. Results are normalized as a fraction of nonsurgical/nonexperimental controls.

### 4.10 SDS‐PAGE and Western blotting

Samples were prepared for SDS‐PAGE and Western blot analysis using complete radioimmunoprecipitation assay (RIPA) lysis buffer (Pierce, #89900) and protease inhibitor cocktail (Pierce, #1860932) as described previously (McGraw et al., 2025; Parajuli et al., 2011). Lysates were centrifuged at 16,000 *xg* for 20 min at 4°C and the supernatant was retained. Protein concentrations were calculated using the BCA Protein Assay kit (Pierce, #23225) and proteins in sample extracts were separated using SDS‐PAGE. Separated proteins were transferred to a PVDF membrane for Western blotting. After the transfer, protein bands were visualized using 1x Red Alert Stain (Millipore, #71078), and the stain was subsequently removed by frequent washes with double‐distilled H_2_O. Membranes were blocked with 5% nonfat milk in TBS‐T and then incubated in primary antibodies as indicated in Table 1. Beta‐actin (Table 1) was used as a loading control for lysates prepared from frozen kidney tissue powder, and VDAC (Table 1) was used as a loading control for mitochondrial extracts. Heat Shock Cognate 71 kDa (Hsc70; antibody used in Table 1) was used as a cytosolic marker for cytosol extracts. Membranes were incubated with horseradish peroxidase (HRP)‐conjugated secondary antibodies as appropriate (Table 1) and washed with TBS‐T to remove background signal. Chemiluminescent imaging was performed using the iBright 1500 imager (Invitrogen, #A44241) and SuperSignal West Pico PLUS Chemiluminescent Substrate (Thermofisher Scientific, #34580). Densitometry was performed in ImageJ software.

### 4.11 Blue‐native PAGE and in‐gel complex V activity

Mitochondrial membrane complexes were extracted from isolated mitochondria (250 μg) using either a 6 g/g digitonin: protein ratio (for visualization of supercomplexes) or a 2.5 g/g lauryl maltoside:protein ratio (for visualization of monomeric complexes). In both cases, extraction was performed in a buffer containing 0.75 M aminocaproic acid and 50 mM Bis‐Tris (pH = 7.0). The mitochondrial extracts were then resolved in a precast Bis‐Tris 3–12% NativePAGE gel (Invitrogen, #BN1001BOX) followed by Western blotting or in‐gel activity.

To detect complex V hydrolytic activity, an in‐gel assay was performed similarly to previous studies (Jha et al., 2016; Timón‐Gómez et al., 2020). First, gels were washed briefly in double‐distilled H_2_O and incubated for 30 min at 22°C in Tris‐Glycine buffer (pH = 8.3) containing 35 mM Tris and 270 mM glycine. Then, gels were incubated for 2 h at 37°C in complex V activity solution containing 35 mM Tris, 270 mM glycine, 14 mM MgSO_4_, 0.2% Pb(NO_3_)_2_ and 8 mM ATP (pH = 8.3). ATP hydrolysis resulted in the development of white lead‐phosphate precipitates, which could be visualized as dark brown bands by incubating the gel in 0.1% ammonium sulfide solution for a few seconds. The reaction was halted using multiple washes with double‐distilled H_2_O and gels were scanned for densitometry analysis.

### 4.12 Cell culture and treatment

Normal rat kidney proximal tubular cells (immortalized cell line NRK‐52E, American Type Culture Collection, #CRL‐1571; sex of cell line is unknown (Shah et al., 2014)) were maintained in Dulbecco's Modified Eagle Medium (DMEM; ATCC, #30–2002) plus 5% fetal calf serum (FCS) and 1% penicillin/streptomycin at 37°C. Data shown are the result of *n* = 3 replicates from 3 independent experiments.

#### 4.12.1 siRNA Transfection

At 50% confluency, NRK cells underwent transient transfection with 100 nM C5 (siC5; siGENOME SMARTpool, Dharmacon, #M‐098499‐01) or 100 nM ATPaf1 (siIF1; siGENOME SMARTpool, Dharmacon, #M‐090981‐00) using siRNA transfection reagent (Dharmacon, #B‐002000‐UB‐100) in OPTI‐MEM (Gibco, #31985–070) with Lipofectamine RNAiMAX (Invitrogen, #13778100) for 48 h at 37°C as previously described (Tobacyk et al., 2019). 100 nM of scrambled siRNA (ON‐TARGET Plus, Dharmacon, #D‐001820‐03) was used as a nontarget control. siRNA concentrations were selected based on the validation of target protein knockdown via Western blotting.

#### 4.12.2 Avacopan Treatment

At 50% confluency, NRK cells were treated with 50 μM AV or vehicle control (VEH) every 24 h to a maximum cumulative dose of 100 μM AV in 48 h for inhibition of the C5a‐C5aR1 axis. The dosing schedule was selected to achieve maximal IF1 induction (visualized via Western blotting; Figure S6D,E) without a severe decrease in cell viability (Figure S6F).

#### 4.12.3 ATP Depletion and Recovery Procedure (Figure 10c)

90–100% confluent NRK cells were washed with PBS and exposed to 10 uM AMA (Sigma, #A8674) in glucose‐free DMEM (Gibco, #A14430‐01) for 2 h at 37°C to deplete cellular ATP as previously described (Cruthirds et al., 2005). After ATP depletion, cells were washed with PBS and returned to normal growth medium (DMEM) for 30 min at 37°C for a period of ATP recovery. During ATP recovery, NRK cells were treated with 5 μM oligomycin (Sigma, #O4876), 6 mM 2‐Deoxy‐D‐glucose (2‐DG; Sigma, #D6134), or vehicle control as outlined in Figure 10c. Post‐ATP recovery, cells were trypsinized and lysed in complete radioimmunoprecipitation assay (RIPA) lysis buffer (Pierce, #89900) and protease inhibitor cocktail (Pierce, #1860932) for downstream applications, including the *ATP Assay*.

### 4.13 Immunocytochemistry

NRK cells were seeded in 8‐chamber slides (Thermofisher Scientific, #154534PK) at a density of 15,000 cells/chamber. Treatment with AV or vehicle control (VEH) was initiated 24 h postseeding. After the treatment period, cells were washed with PBS‐T and fixed in 4% formaldehyde for 15 min at 22°C. Permeabilization was carried out using 0.1% Triton X‐100 and 0.1% sodium citrate for 2 min at 4°C. Permeabilized cells were blocked with 5% normal goat serum in PBS‐T for 1 h at 22°C before incubation with primary antibodies as indicated in Table 1. After primary antibody incubation, cells were incubated with Alexa Fluor‐conjugated secondary antibodies as shown in Table 1. Nuclei were stained with 300 nM DAPI, and cells were washed multiple times with PBS before mounting of the chamber slide using ProLong Gold Antifade Mountant with DAPI (Invitrogen, #P36931).

### 4.14 Confocal microscopy and colocalization

After immunocytochemical labelling, mitochondrial proteins were visualized using an LSM 880 confocal microscope 63x oil objective plus Airyscan at 100x magnification. Confocal z‐stacks were generated and processed using ZEISS Zen Black system 2.3 software (Carl Zeiss Microscopy, White Plains, New York). 20 z‐stacks/field from 5 representative fields were collected from three independent cultures (experiments) for each comparison. Colocalization analysis was performed in ZEISS Zen Black system 2.3 software (Carl Zeiss Microscopy, White Plains, New York) using the Manders' coefficient method (Manders et al., 1993). Colocalization coefficients were weighted to account for areas of low pixel intensity and averaged per ROI. Data points reflect independent experiments.

### 4.15 JC‐1 assay

NRK cells were seeded in 35 mm culture dishes or 96‐well plates at a density of 100,000 cells/dish or 10,000 cells/well, respectively. Transfection and drug treatments, when applicable, were initiated 24 h postseeding. After the treatment period, cells were washed twice with prewarmed PBS and were incubated with 2.5 μM JC‐1 dye (Invitrogen, #T3168) at 37°C for 30 min. 35 mm dishes were then washed twice with prewarmed PBS and the live cells were imaged immediately using a Nikon Eclipse E800 microscope in Nikon Elements software. 96‐well plates were washed twice with prewarmed PBS and the fluorescent signal was immediately quantified using a BioTek microplate reader (Agilent Technologies, Santa Clara, California). Red fluorescence of J‐aggregates was detected at an emission maximum of 590 nm, and green fluorescence of J‐monomers was detected at an emission maximum of 530 nm. The mitochondrial membrane potential was quantified as the red aggregate: green monomer ratio.

### 4.16 Statistical analysis

Statistical analysis was performed in GraphPad Prism software (version 10) and data are presented as the mean ± SD. Normal distribution of the data was confirmed using the Shapiro–Wilk normality test. As appropriate, data were assessed using a one‐way or two‐way ANOVA with a Tukey's posthoc test for multiple comparisons. For the skewed data, the Mann–Whitney *U* test was performed to compare the difference between the two groups. *p*‐values <0.05 were utilized to define statistical significance. Graphical figures were generated in GraphPad Prism software.

## AUTHOR CONTRIBUTIONS

**Madison McGraw:** Data curation; formal analysis; investigation; methodology; visualization. **Amod Sharma:** Methodology. **Dinesh Bhattarai:** Formal analysis; methodology. **Neriman Gokden:** Formal analysis; visualization. **LeeAnn Macmillan‐Crow:** Supervision. **Nirmala Parajuli:** Conceptualization; data curation; funding acquisition; resources; supervision; visualization.

## FUNDING INFORMATION

National Institute of Diabetes and Digestive and Kidney Diseases (R01 DK123264), UAMS Barton Pilot Award (17‐DN‐08), and American Heart Association (AHA) (19TPA34850057).

## CONFLICT OF INTEREST STATEMENT

The authors declare no conflicts of interest.

## ETHICS STATEMENT

Animal studies were approved by an Institutional Animal Care and Use Committee (IACUC) at the University of Arkansas for Medical Sciences (Approval No. IPROTO202400000031).

## INFORMED CONSENT STATEMENT

Not applicable (no human studies).

## Supporting information


Data S1.

**Figure S1.** Effect of C5‐C5aR1 on renal mitochondrial protein levels. Renal cytosol and mitochondria fractions from male wild‐type (C5^+/+^), homozygous (C5^−/−^), and AV‐treated (C5^+/+^+AV) rats (*n* = 4 per group) were isolated and lysed with RIPA after sham or IRI surgery. 10 μg of RIPA lysate per sample was resolved through SDS‐PAGE (4%–12% gradient gel) and Western blotting was performed using antibodies as outlined in Table 1. (A) Representative Western blots of isolated cytosol and mitochondria fractions comparing C5^+/+^ and C5^−/−^ groups. Corresponding bar graphs reflect the densitometric ratio of proteins in the (B) mitochondrial and (C) cytosolic fractions, respectively. Data are shown as the mean +/− SD, *n* = 4. (D) Representative Western blots of isolated cytosol and mitochondria fractions comparing the C5^+/+^ and AV‐treated (C5^+/+^+AV) groups. Corresponding bar graphs reflect the densitometric ratio of proteins in the (B) mitochondrial and (C) cytosolic fractions. Data are shown as the mean +/− SD, *n* = 4.
**Figure S2:** Effect of C5‐C5aR1 on the levels of renal mitochondria electron transport complexes. Renal mitochondrial membranes from male wild‐type (C5^+/+^), homozygous (C5^−/−^), and AV‐treated (C5^+/+^+AV) rats were isolated and solubilized with 10% lauryl maltoside after sham or IRI surgery. 10 μg of solubilized protein per sample was resolved using BN‐PAGE for downstream Coomassie staining or Western blotting (antibodies were employed as shown in Table 1) applications. (A‐B) Representative Coomassie‐stained gels of isolated mitochondrial membrane proteins are shown (*n* = 4 per group) and the bands reflecting the mitochondrial electron transport complexes are identified using their respective molecular weights. For C5^+/+^ and C5^−/−^ experimental group comparisons, representative Western blots depict the levels of mitochondrial electron transport complexes (C) I, (E) II, (G) III, (I) IV, and (K) V, respectively. Corresponding bar graphs (D, F, H, J, L) reflect the densiometric ratio of each electron transport complex band to the total protein per gel lane. Data are shown as the mean +/− SD, *n* = 4.
**Figure S3:** In‐gel complex V activity. Renal mitochondrial membranes from male C5^+/+^, C5^−/−^, and C5^+/+^+AV rats were isolated and solubilized with 10% lauryl maltoside after sham or IRI surgery. 10 μg of solubilized protein per sample was resolved using BN‐PAGE for downstream in‐gel complex V activity assays. (A) For C5^+/+^ and C5^−/−^ group comparisons, a representative gel depicting the white precipitate band representing ATP hydrolytic activity is shown. (B) For C5^+/+^ and C5^+/+^+AV group comparisons, the representative gel depicting the white precipitate band representing ATP hydrolytic activity is shown.
**Figure S4:** Effect of C5‐C5aR1 on the native organization of renal mitochondrial membrane complexes. Renal mitochondrial membranes from male wild‐type (C5^+/+^), homozygous (C5^−/−^), and AV‐treated (C5^+/+^+AV) rats were isolated and solubilized with 10% digitonin after sham or IRI surgery. 10 μg of solubilized protein per sample was resolved using BN‐PAGE for downstream Coomassie staining or Western blotting (antibodies were employed as shown in Table 1) applications. (A‐B) Representative Coomassie‐stained gels of isolated mitochondrial membrane proteins are shown (*n* = 4 per group). For C5^+/+^ and C5^−/−^ experimental group comparisons, representative Western blots depict supramolecular structures containing electron transport complexes (C) I, (E) II, (G) III, (I) IV, and (K) V, respectively. Corresponding bar graphs (D, F, H, J, L) reflect the densiometric ratio of so‐called “supercomplexes” to the total protein per gel lane. Data are shown as the mean +/− SD, *n* = 4. For C5^+/+^ and AV‐treated (C5^+/+^+AV) experimental group comparisons, representative Western blots display supercomplexes containing the electron transport complexes (M) I, (O) II, (Q) III, (S) IV, and (U) V, respectively. Corresponding bar graphs (N, P, R, T, V) indicate the densitometric ratio of supercomplexes to the total protein per gel lane. Data are shown as the mean +/− SD, *n* = 4. Throughout this figure, the respective representative images and densitometric ratios of electron transport complexes solubilized with 10% lauryl maltoside are shown for the purpose of comparison.
**Figure S5:** C5‐C5aR1 inhibition mediates IF1 protein localization in kidney tissue. Male wild‐type (C5^+/+^) and homozygous (C5^−/−^) rats were exposed to a surgical IRI model of 1 h bilateral renal ischemia and 24 h reperfusion. To isolate C5aR1‐specific effects, a group of C5^+/+^ rats were dosed with 30 mg/kg AV i.p. 1 h prior to surgery. Sham surgery (right nephrectomy) was performed to serve as a control. Kidneys were isolated after IRI and processed for immunohistochemical analysis. (A) Representative micrographs (*n* = 4 per group) from formalin‐fixed sections of sham kidneys immunohistochemically stained for IF1.
**Figure S6:** Validation of IF1 induction in normal rat kidney (NRK) proximal tubular cells. (A‐C) NRK cells (*n* = 3 individual experiments) were transiently transfected with siRNA targeted to C5 (siC5) or a scrambled control. 48 h posttransfection, cells were lysed with RIPA and processed for SDS‐PAGE. (A) Representative Western blot of NRK cell lysate probed with C5 and IF1 antibodies as depicted in Table 1. Corresponding bar graphs show the densitometry analysis of (B) C5 and (C) IF1 protein levels in cell lysates, respectively. Data are shown as the mean +/− SD, *n* = 3. (D‐E) NRK cells (*n* = 3 individual experiments) were treated with AV (50–200 μM cumulative dose) or an equivalent volume of vehicle (VEH) over 48 h. After the treatment period, cells were lysed with RIPA and processed for SDS‐PAGE. (A) Representative Western blot of NRK cell lysate probed with IF1 antibody. A corresponding bar graph displays (E) densitometry analysis of IF1 protein levels in VEH‐ or AV‐treated cell lysates. Data are shown as the mean +/− SD, *n* = 3. (F) NRK cells (*n* = 3 individual experiments) were treated with VEH or AV (1, 10, or 100 μM dose) and the cell viability was measured using an ATP‐based assay. Data are shown as the mean +/− SD, *n* = 3.
**Figure S7:** IF1 colocalizes to the inner mitochondrial membrane. VEH‐treated NRK cells were immunocytochemically labeled with IF1 (red) or other mitochondrial protein antibodies (green) as outlined in Table 1. DAPI was used to label cell nuclei. (A) Representative micrographs reflect the immunofluorescent signal in NRK cells, imaged in z‐stacks using an LSM 880 confocal microscope plus Airyscan (100x magnification). Scale bar = 100 pixels. Corresponding bar graphs (B‐C) display the quantification of fractional colocalization, as represented by weighted Mander's Colocalization Coefficients. M_Green_ = the fractional colocalization of the green fluorescent signal overlapping with red fluorescent signal, and M_Red_ = the fractional colocalization of the red fluorescent signal (IF1) overlapping with green fluorescent signal. Data are shown as the mean +/− SD, *n* = 3 individual experiments.

## Data Availability

All data associated with this study are present in the paper. Data will be made available upon reasonable request.
