# Supplementary material for: Novel interactions between the C5‐C5aR1 Axis and IF1: Implications for kidney mitochondrial physiology and ischemia–reperfusion injury
Source: Physiol Rep. 2026 May 29;14(11):e70942. doi: 10.14814/phy2.70942 (PMC13239135; doi:10.14814/phy2.70942)
Supplement: Supplementary file 1 — Data S1. [file PHY2-14-e70942-s001.pdf]

Supplemental Methods

Cell Viability Assay. Cell viability was measured using a CellTiter-Glo ATP Measurement kit (Promega, #G7570) according to the manufacturer's instructions[123]. Briefly, 96-well plates were seeded with 10,000 NRK cells per well in warmed DMEM (37˚C) plus 10% FCS. After a 24 hr growth period, NRK cells were treated with 1 uM, 10 uM, or 100 uM Avacopan (**AV**) or a DMSO vehicle control (**VEH**). Additional controls included NRK cells that received no treatment (**UnTx**) and NRK cells receiving no treatment except wash steps comparable to the treatment groups (**Sham**). After the 24 hr treatment period, cells were lysed within wells using a passive lysis buffer and were analyzed for luciferase activity using the manufacturer's Bright-Glo luciferase assay system. The ATP-based cell viability was quantified as luminescence using a BioTek microplate reader (Agilent Technologies, Santa Clara, California).
