# Supplementary material for: Novel interactions between the C5‐C5aR1 Axis and IF1: Implications for kidney mitochondrial physiology and ischemia–reperfusion injury
Source: Physiol Rep. 2026 May 29;14(11):e70942. doi: 10.14814/phy2.70942 (PMC13239135; doi:10.14814/phy2.70942)

Supplemental Figure 1

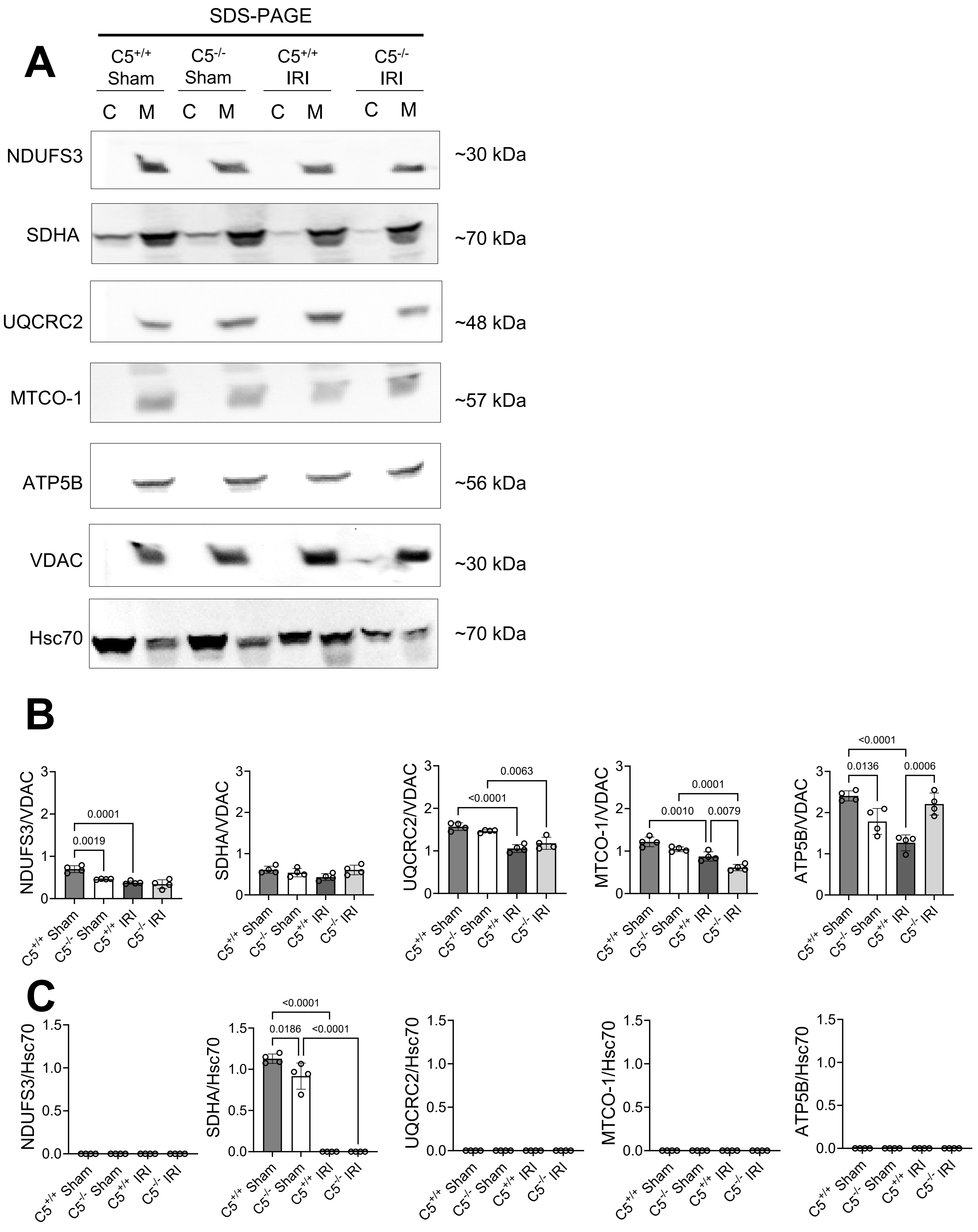

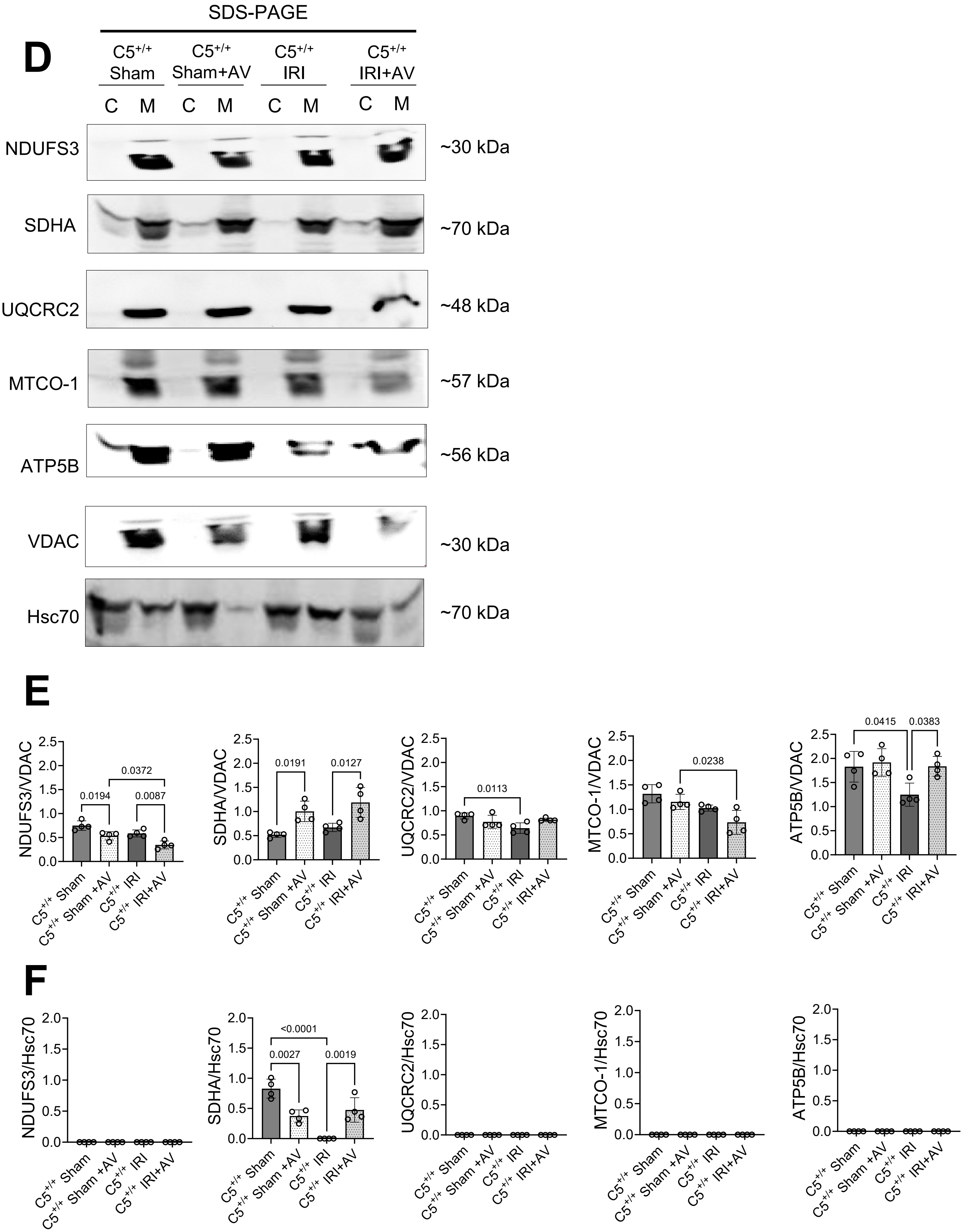

D
SDS-PAGE
C5+/+ Sham
C5+/+ Sham+AV
C5+/+ IRI
C5+/+ IRI+AV
C M C M C M C M
NDUFS3 ~30 kDa
SDHA ~70 kDa
UQCRC2 ~48 kDa
MTCO-1 ~57 kDa
ATP5B ~56 kDa
VDAC ~30 kDa
Hsc70 ~70 kDa
E
NDUFS3/VDAC
0.0194
0.0372
0.0087
SDHA/VDAC
0.0191
0.0127
UQCRC2/VDAC
0.0113
MTCO-1/VDAC
0.0238
ATP5B/VDAC
0.0415
0.0383
C5+/+ Sham
C5+/+ Sham +AV
C5+/+ IRI
C5+/+ IRI+AV
F
NDUFS3/Hsc70
SDHA/Hsc70
<0.0001
0.0027
0.0019
UQCRC2/Hsc70
MTCO-1/Hsc70
ATP5B/Hsc70

Supplemental Figure 2

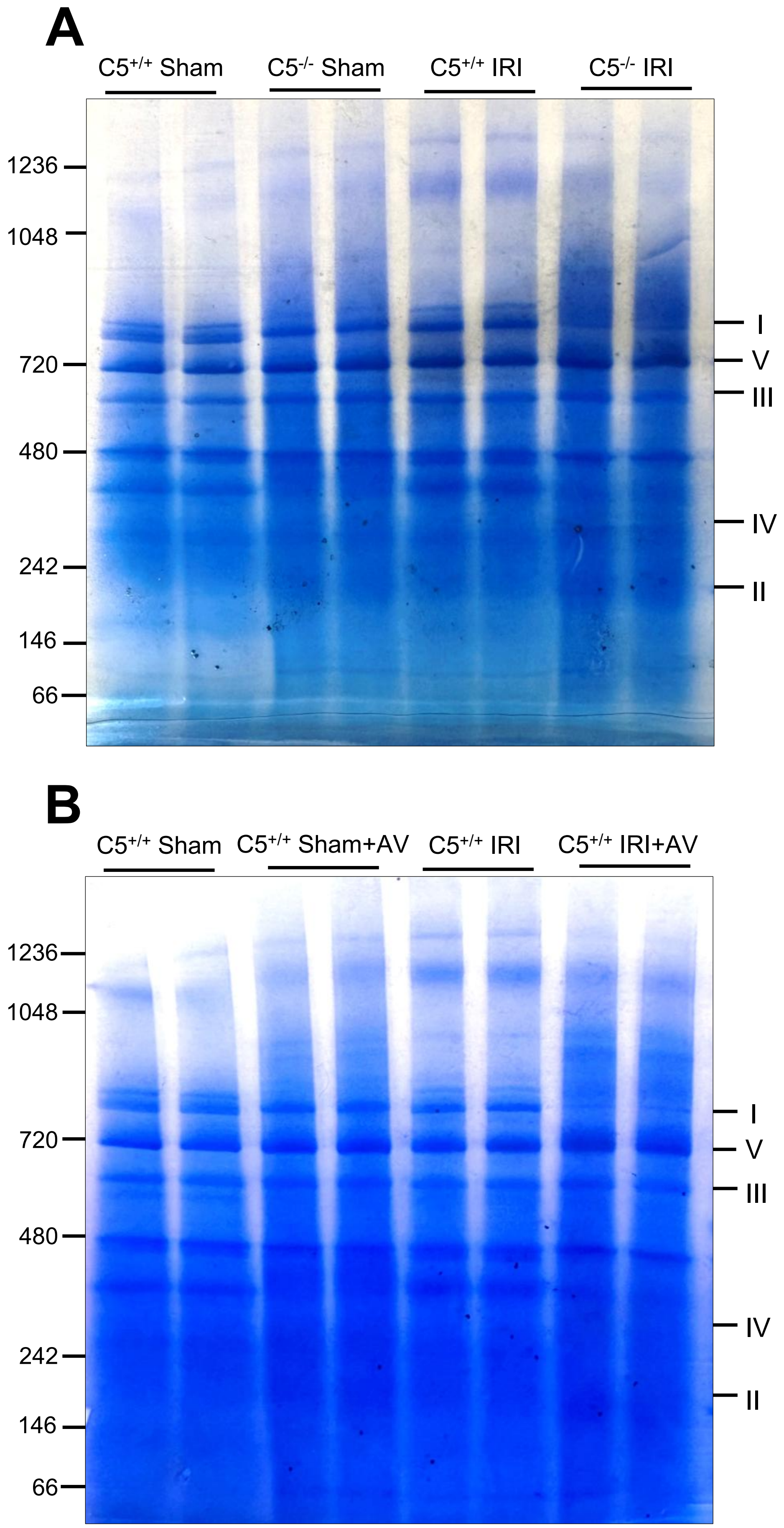

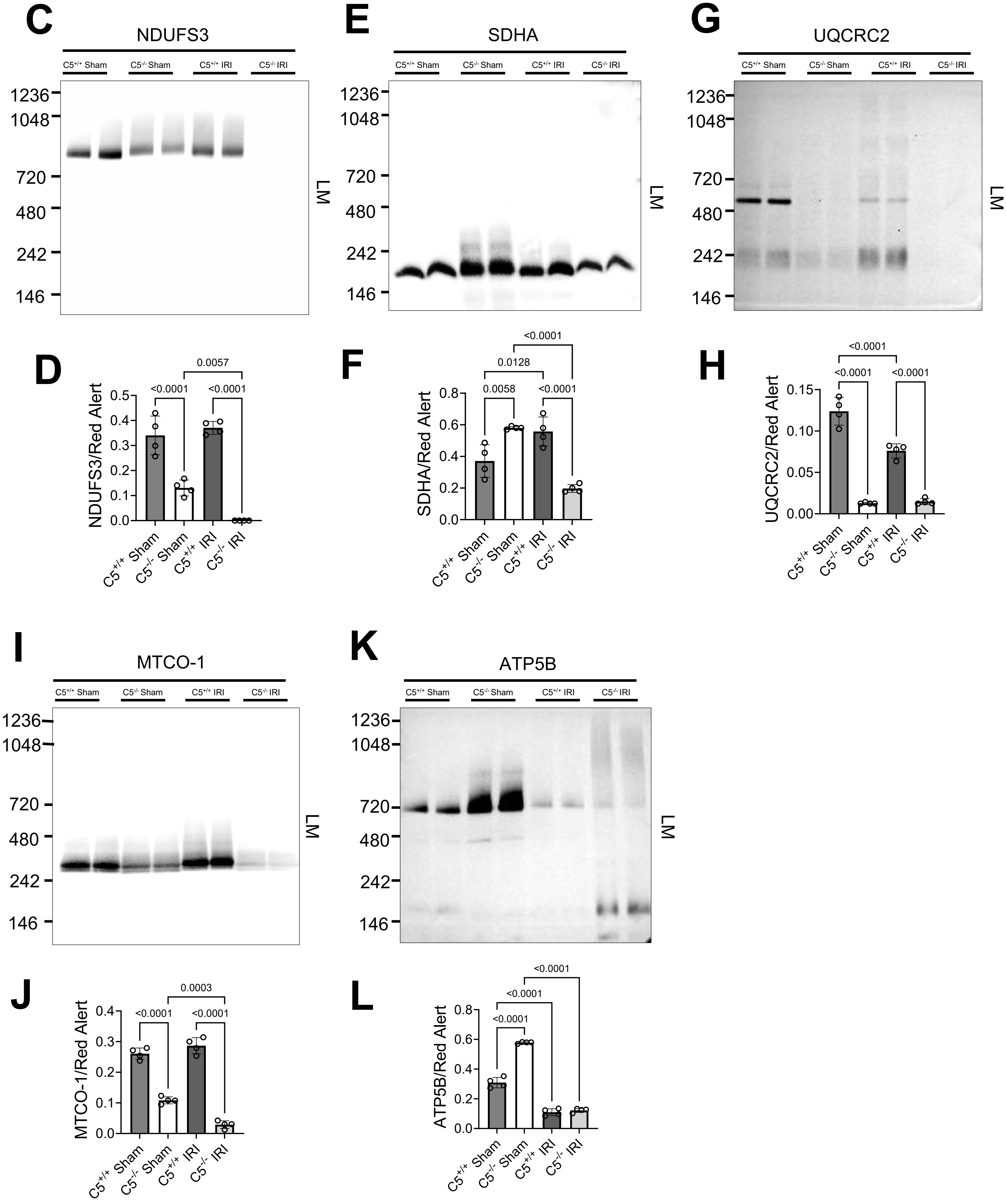
C
NDUFS3
C5+/+ Sham
C5-/- Sham
C5+/+ IRI
C5-/- IRI
1236
1048
720
480
242
146
LM
E
SDHA
G
UQCRC2
D
NDUFS3/Red Alert
0.0057
<0.0001
<0.0001
F
SDHA/Red Alert
<0.0001
0.0128
0.0058
<0.0001
H
UQCRC2/Red Alert
<0.0001
<0.0001
<0.0001
I
MTCO-1
K
ATP5B
J
MTCO-1/Red Alert
0.0003
<0.0001
<0.0001
L
ATP5B/Red Alert
<0.0001
<0.0001
<0.0001

Supplemental Figure 3

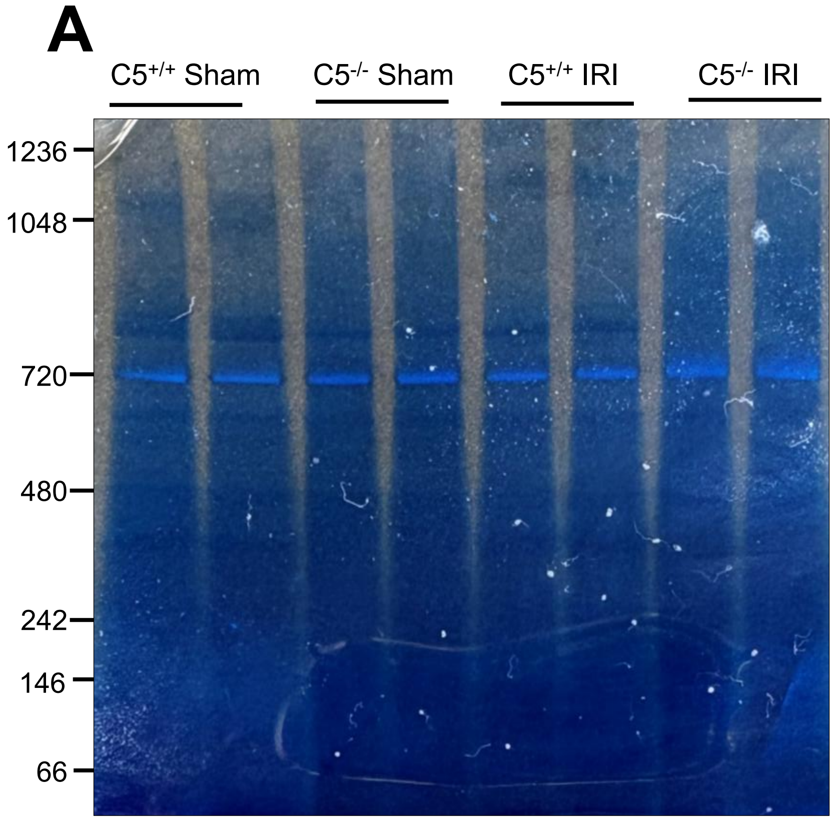

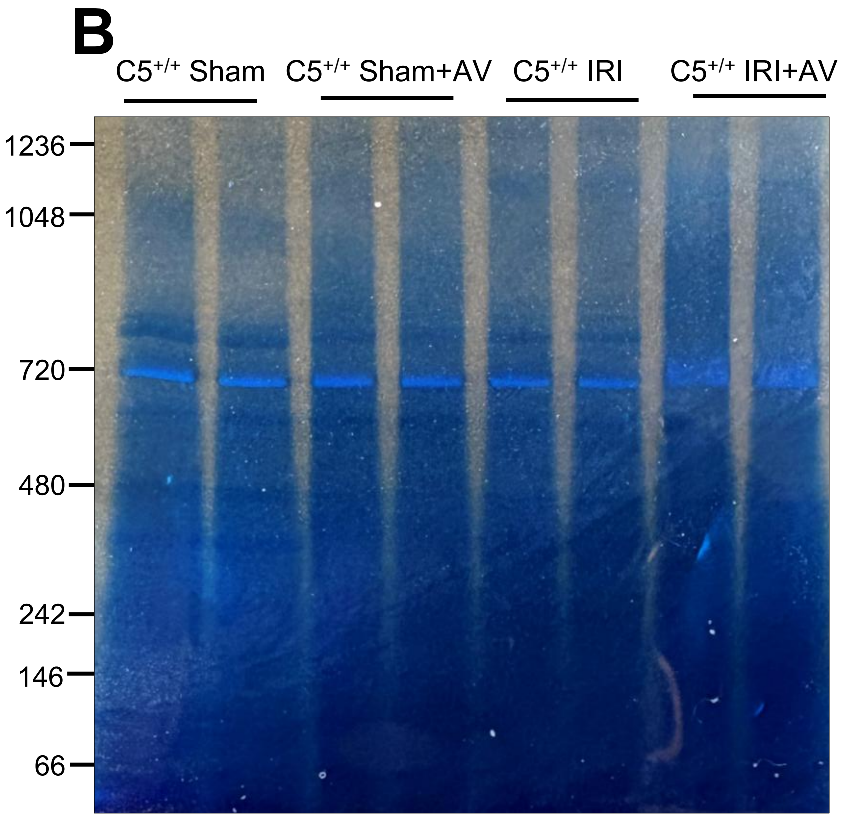

Supplemental Figure 4

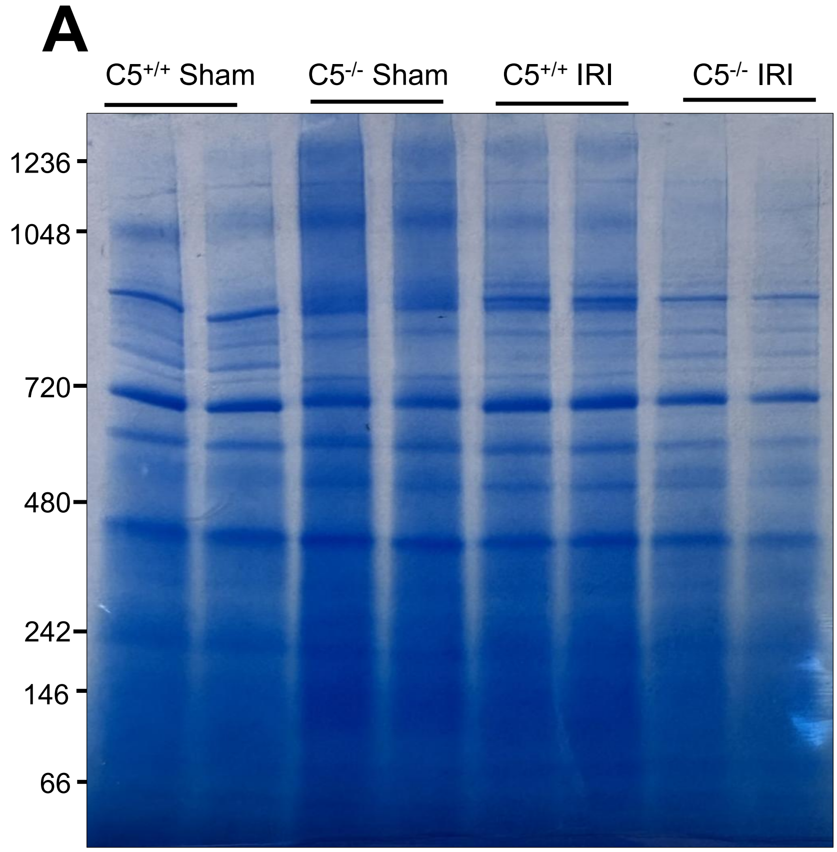

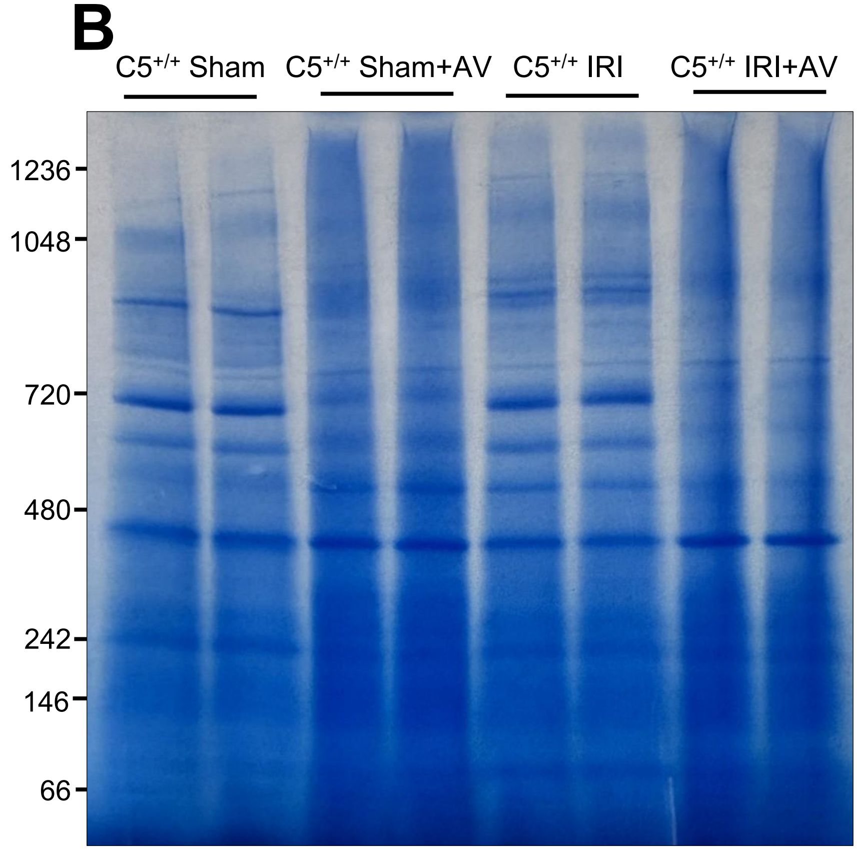

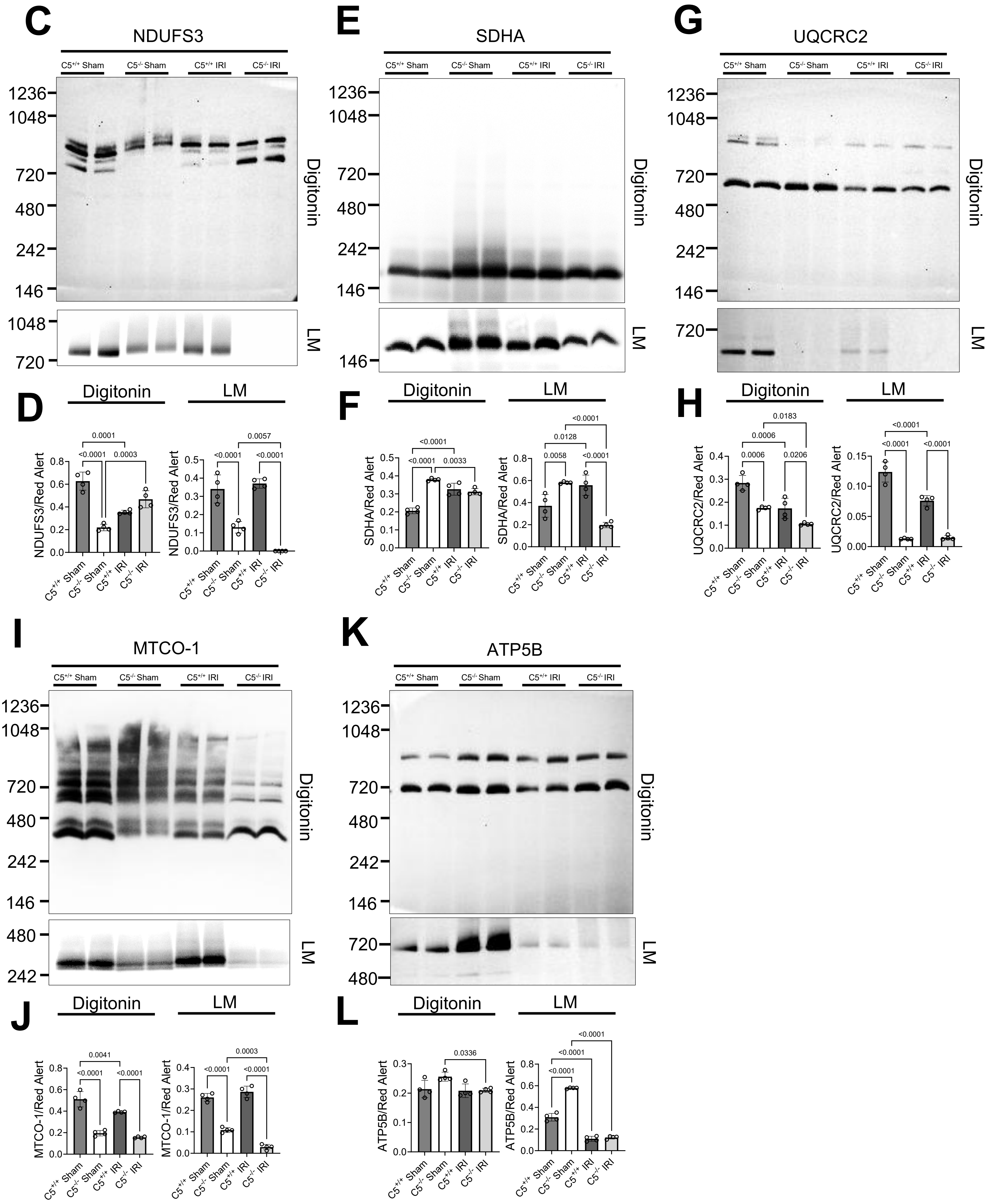

C
NDUFS3
C5+/+ Sham
C5-/- Sham
C5+/+ IRI
C5-/- IRI
1236
1048
720
480
242
146
Digitonin
LM
D
Digitonin
LM
NDUFS3/Red Alert
0.0001
<0.0001
0.0003
0.0057
<0.0001
<0.0001
E
SDHA
F
SDHA/Red Alert
<0.0001
<0.0001
0.0033
<0.0001
0.0128
0.0058
<0.0001
G
UQCRC2
H
UQCRC2/Red Alert
0.0183
0.0006
0.0006
0.0206
<0.0001
<0.0001
<0.0001
I
MTCO-1
J
MTCO-1/Red Alert
0.0041
<0.0001
<0.0001
0.0003
<0.0001
<0.0001
K
ATP5B
L
ATP5B/Red Alert
0.0336
<0.0001
<0.0001
<0.0001

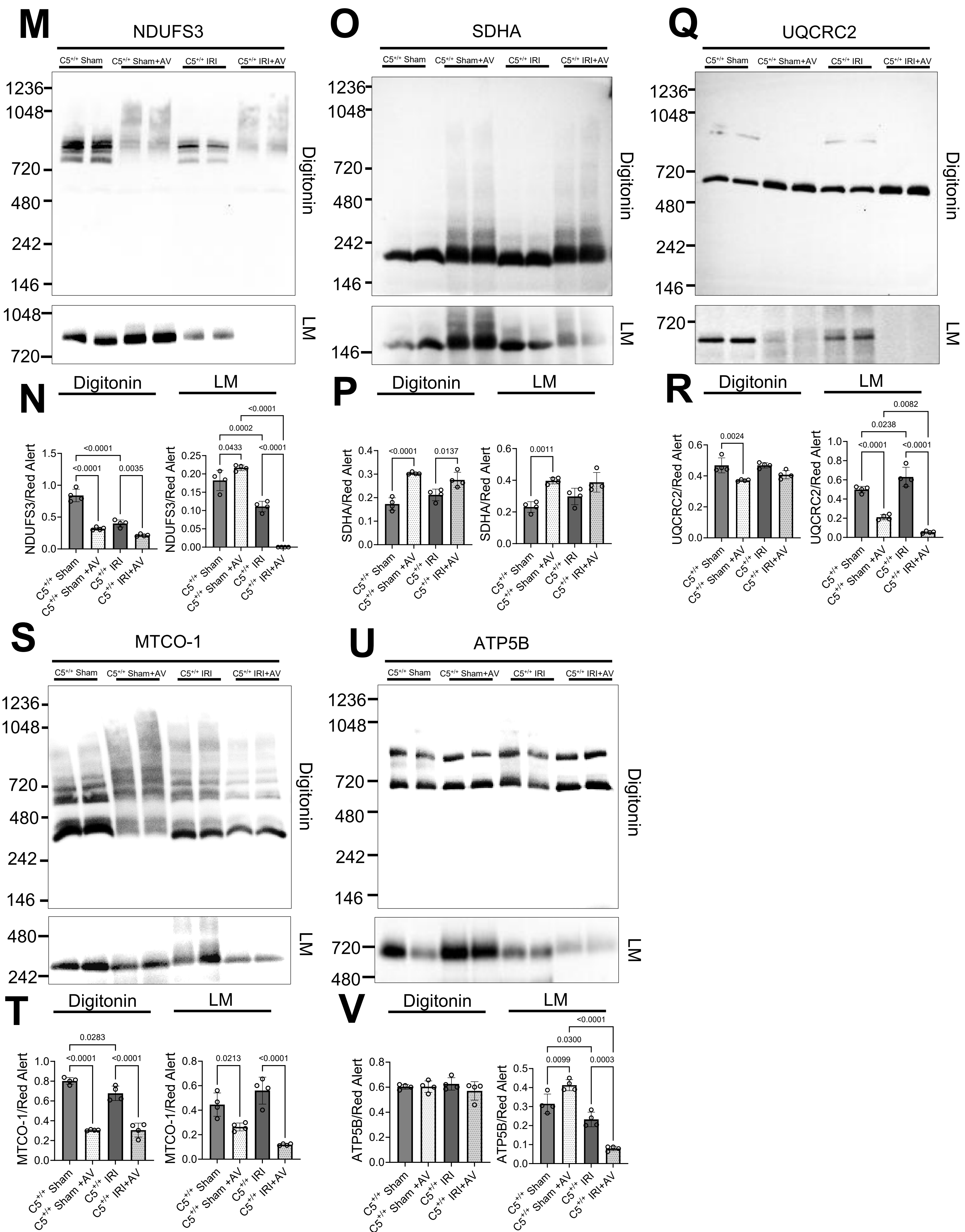
M
NDUFS3
C5+/+ Sham
C5+/+ Sham+AV
C5+/+ IRI
C5+/+ IRI+AV
1236
1048
720
480
242
146
Digitonin
LM
O
SDHA
Q
UQCRC2
N
Digitonin
LM
NDUFS3/Red Alert
<0.0001
0.0035
0.0433
0.0002
P
SDHA/Red Alert
0.0137
0.0011
R
UQCRC2/Red Alert
0.0024
0.0238
0.0082
S
MTCO-1
U
ATP5B
T
MTCO-1/Red Alert
0.0283
0.0213
V
ATP5B/Red Alert
0.0300
0.0099
0.0003
C5+/+ Sham +AV
C5+/+ IRI+AV

Supplemental Figure 5

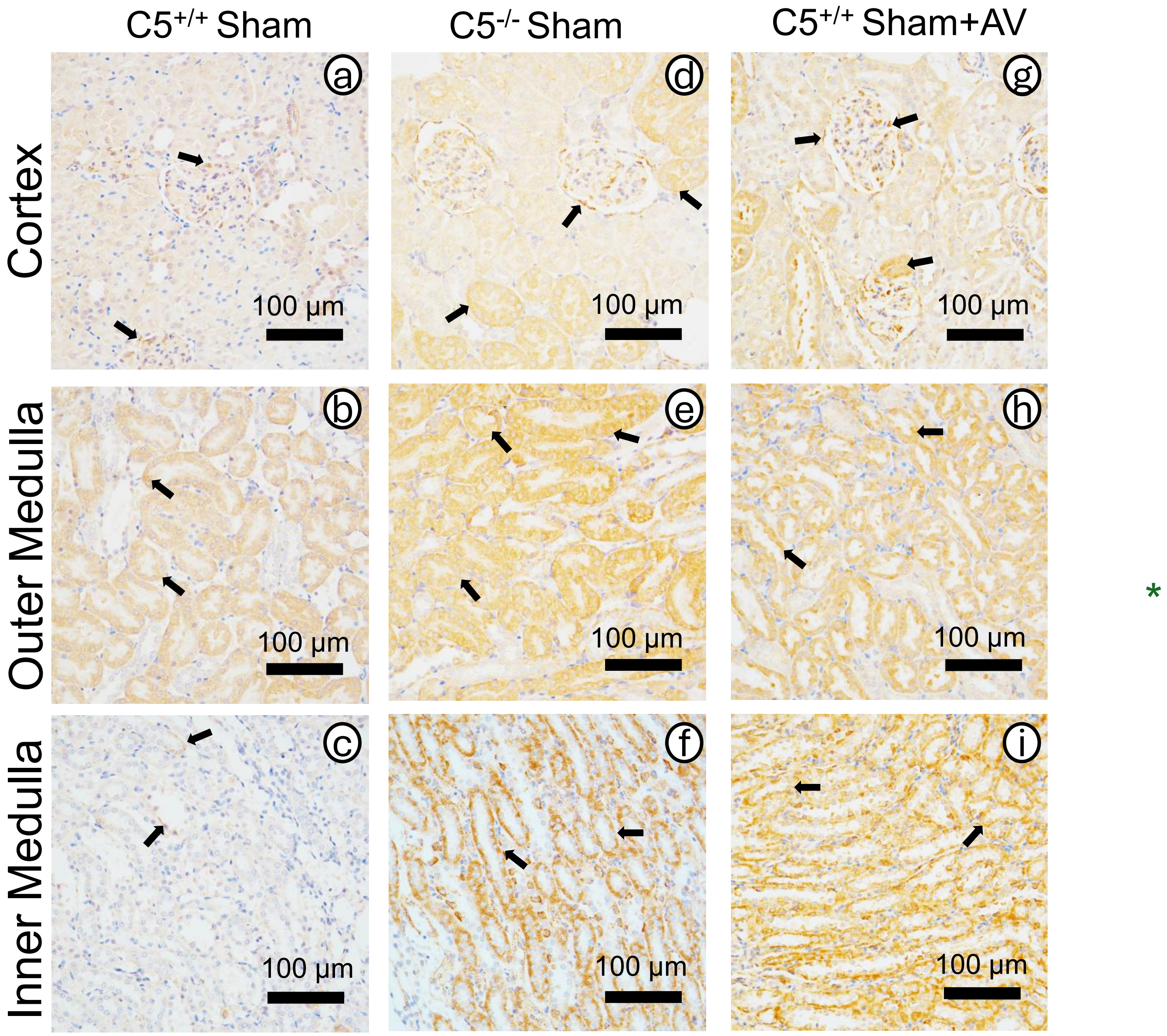

Supplemental Figure 6

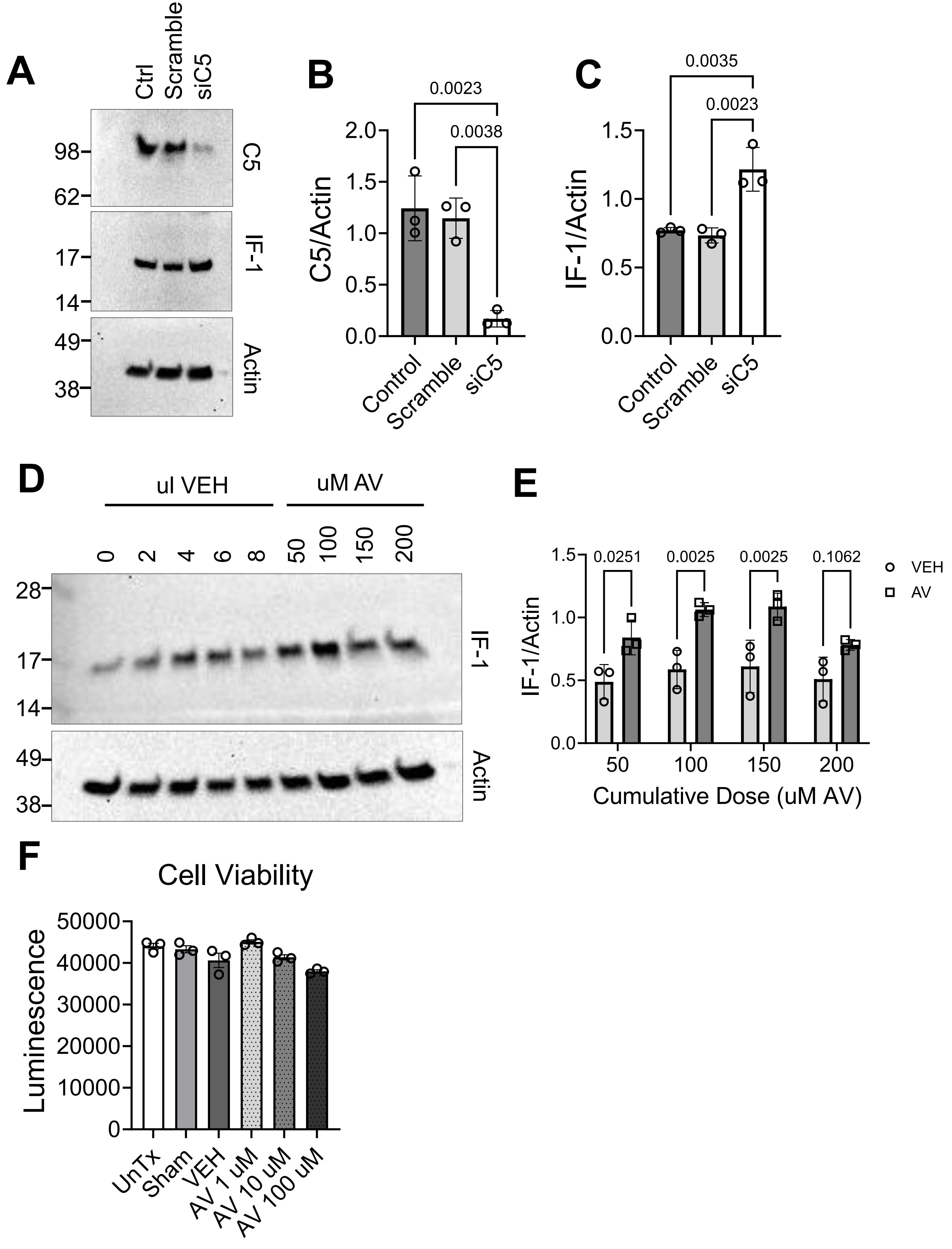

Supplemental Figure 7

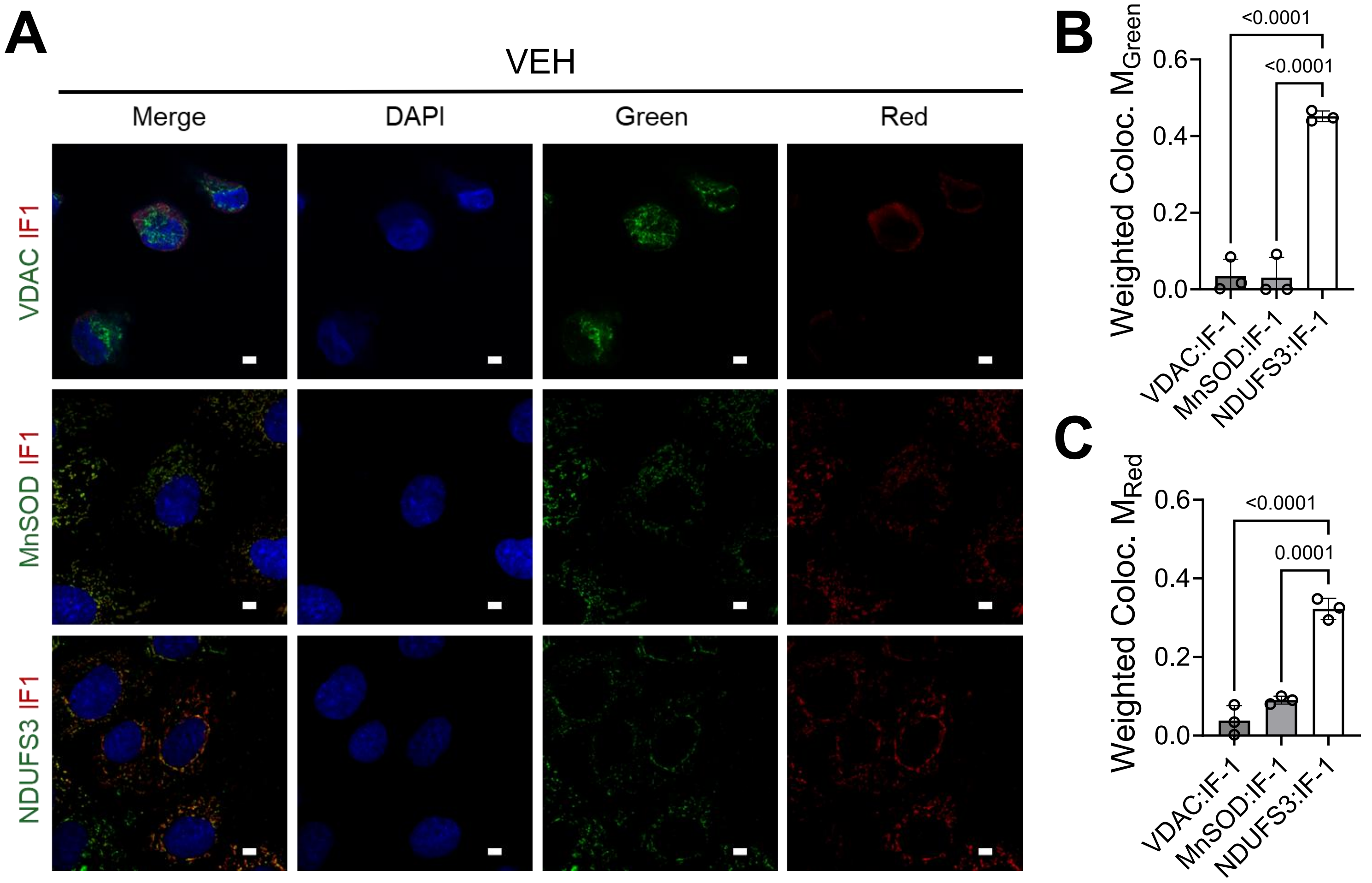

Supplement: Supplementary file 2 — Figure S1. Effect of C5‐C5aR1 on renal mitochondrial protein levels. Renal cytosol and mitochondria fractions from male wild‐type (C5+/+), homozygous (C5−/−), and AV‐treated (C5+/++AV) rats (n = 4 per group) were isolated and lysed with RIPA after sham or IRI surgery. 10 μg of RIPA lysate per sample was resolved through SDS‐PAGE (4%–12% gradient gel) and Western blotting was performed using antibodies as outlined in Table 1. (A) Representative Western blots of isolated cytosol and mitochondria fractions comparing C5+/+ and C5−/− groups. Corresponding bar graphs reflect the densitometric ratio of proteins in the (B) mitochondrial and (C) cytosolic fractions, respectively. Data are shown as the mean +/− SD, n = 4. (D) Representative Western blots of isolated cytosol and mitochondria fractions comparing the C5+/+ and AV‐treated (C5+/++AV) groups. Corresponding bar graphs reflect the densitometric ratio of proteins in the (B) mitochondrial and (C) cytosolic fractions. Data are shown as the mean +/− SD, n = 4. Figure S2: Effect of C5‐C5aR1 on the levels of renal mitochondria electron transport complexes. Renal mitochondrial membranes from male wild‐type (C5+/+), homozygous (C5−/−), and AV‐treated (C5+/++AV) rats were isolated and solubilized with 10% lauryl maltoside after sham or IRI surgery. 10 μg of solubilized protein per sample was resolved using BN‐PAGE for downstream Coomassie staining or Western blotting (antibodies were employed as shown in Table 1) applications. (A‐B) Representative Coomassie‐stained gels of isolated mitochondrial membrane proteins are shown (n = 4 per group) and the bands reflecting the mitochondrial electron transport complexes are identified using their respective molecular weights. For C5+/+ and C5−/− experimental group comparisons, representative Western blots depict the levels of mitochondrial electron transport complexes (C) I, (E) II, (G) III, (I) IV, and (K) V, respectively. Corresponding bar graphs (D, F, H, J, L) reflect the densiome [file PHY2-14-e70942-s002.pdf]
